# Epigenetic Silencing of CYYR1 Promotes Lung Adenocarcinoma Progression Through TRIM21‐Dependent Regulation of HMMR Stability

**DOI:** 10.1002/advs.77890

**Published:** 2026-10-11

**Authors:** Fengsheng Dai, Jingyi Tang, Huan Rong, Yu Wang, Xiangyi Zhou, Can Xu, Jianhua Wang, Lili Li, Yue Wu, Fei Deng, Dan Yang, Xiao Yang, Renyan Li, Yongzhong Wu, Dongxi Xiang, Tingxiu Xiang

**Affiliations:** ^1^ Chongqing Key Laboratory for the Mechanism and Intervention of Cancer Metastasis Chongqing University Cancer Hospital Chongqing University Chongqing China; ^2^ School of Pharmacy and Bioengineering Chongqing University of Technology Chongqing China; ^3^ Institute of Image Processing and Pattern Recognition Shanghai Jiao Tong University Shanghai China; ^4^ Department of Clinical Oncology State Key Laboratory of Translational Oncology Sir YK Pao Centre for Cancer The Chinese University of Hong Kong Hong Kong SAR China; ^5^ Graduate School of Biomedical Engineering Faculty of Engineering University of New South Wales Sydney New South Wales Australia; ^6^ State Key Laboratory of Systems Medicine for Cancer Shanghai Cancer Institute Shanghai Key Laboratory of Cancer System Regulation and Clinical Translation Department of Biliary‐Pancreatic Surgery, Department of Gastrointestinal Surgery, Renji Hospital Shanghai Jiao Tong University School of Medicine Shanghai China

**Keywords:** CDC20, CYYR1, HMMR, lung adenocarcinoma, TRIM21, tumor suppression, ubiquitination

## Abstract

Lung adenocarcinoma (LUAD) remains a leading cause of cancer‐related mortality. Integrative multi‐omics and machine‐learning analyses across independent cohorts identified CYYR1 as a frequently downregulated transmembrane protein in LUAD, with low expression associated with poor prognosis and promoter hypermethylation. CYYR1 restoration suppressed LUAD cell proliferation, migration, invasion, tumor growth, and metastasis. Mechanistically, CYYR1 facilitated TRIM21‐dependent ubiquitination and proteasomal degradation of HMMR, thereby reducing CDC20‐associated oncogenic signaling. Biochemical and cellular analyses supported this CYYR1–TRIM21–HMMR regulatory relationship, while a patient‐derived LUAD organoid model provided proof‐of‐concept evidence that CYYR1 restoration inhibited organoid growth and reduced HMMR, CDC20, and Ki67 expression. CYYR1 restoration also increased cisplatin sensitivity. Genistein treatment was associated with CDC20 modulation and enhanced cisplatin response, and cellular thermal shift assays provided evidence consistent with CDC20 engagement, although CDC20 dependence of the Genistein response remains unproven. Collectively, these findings identify CYYR1 as an epigenetically dysregulated tumor‐suppressive factor that regulates HMMR stability through a TRIM21‐dependent mechanism and reveal a potential therapeutic vulnerability in CYYR1‐deficient LUAD.

## Graphical abstract

CYYR1 as an epigenetically dysregulated tumor‐suppressive factor that influences oncoprotein stability through TRIM21‐dependent HMMR regulation and reveals a potential therapeutic vulnerability in CYYR1‐deficient LUAD. The graphical illustration was created using FigDraw (www.figdraw.com).

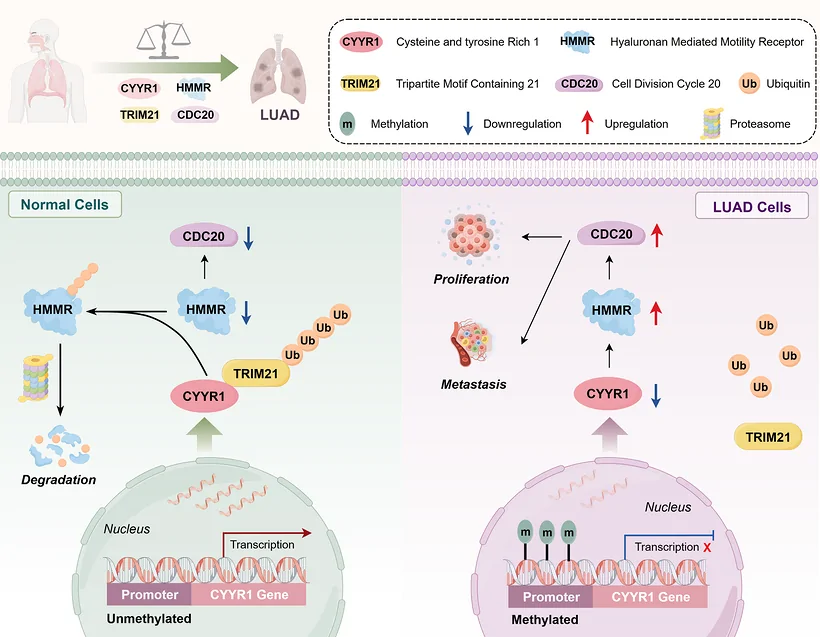

## 1 Introduction

Lung adenocarcinoma (LUAD) remains a leading cause of cancer‐related mortality worldwide, with poor clinical outcomes largely attributed to tumor progression, metastatic dissemination, and therapeutic resistance [1, 2]. The limited efficacy of current treatment strategies highlights the urgent need to identify novel molecular drivers of LUAD pathogenesis and uncover new therapeutic vulnerabilities [3].

Cysteine‐tyrosine‐rich protein 1 (CYYR1) is an evolutionarily conserved, single‐pass transmembrane protein encoded on chromosome 21 (21q21.2) [4, 5, 6]. Beyond its reported role in lipid metabolism [7] and certain non‐malignant conditions [8, 9], CYYR1 has begun to emerge in an oncological context. For instance, its promoter methylation status holds prognostic value in neuroblastoma, and its expression is linked to favorable outcomes in breast cancer, suggesting a potential tumor‐suppressive role [10, 11]. However, whether CYYR1 contributes to LUAD progression and whether it participates in tumor‐associated protein regulation remain unknown.

Hyaluronan‐mediated motility receptor (HMMR) is a multifunctional protein involved in spindle assembly and hyaluronic acid signaling, and it drives tumor progression by regulating metastasis, stemness, and endoplasmic reticulum stress across diverse cancer types [12, 13, 14, 15, 16, 17, 18]. Although HMMR overexpression has been associated with poor prognosis and metastatic behavior in LUAD and NSCLC, the upstream mechanisms controlling HMMR protein stability remain incompletely defined [19].

Here, we report that CYYR1 is epigenetically silenced via promoter hypermethylation in LUAD and exerts tumor‐suppressive activity, with reduced expression associated with unfavorable patient prognosis. Mechanistically, we identify a previously unrecognized regulatory pathway in which CYYR1 facilitates TRIM21‐dependent ubiquitination and proteasomal degradation of the oncoprotein HMMR. This regulatory cascade suppresses CDC20‐associated oncogenic signaling and contributes to inhibition of LUAD malignant phenotypes. We further examined CYYR1‐associated pathway regulation in a patient‐derived LUAD organoid model as a proof‐of‐concept extension of the cell‐based findings and explored its potential therapeutic implications. Our study identifies CYYR1 as a potential prognostic biomarker and reveals a potential therapeutic vulnerability associated with CYYR1 deficiency in LUAD.

## 2 Materials and Methods

### 2.1 Cell Lines and Clinical Specimens

The human bronchial epithelial cell line BEAS‐2B and a panel of lung cancer cell lines (A549, H1299, H358, PC‐9, PC‐9/GR, H1975, CAL12T, H2122, H1651, CHAGO‐K‐1, SW1271, H446, and H460) were obtained from commercially available certified repositories and cultured according to the recommended protocols. BEAS‐2B cells were maintained in DMEM, whereas lung cancer cell lines were cultured in RPMI‐1640 medium, both supplemented with 10% fetal bovine serum (Viva cell, C04‐001) and 1% penicillin–streptomycin (Gibco, 15140122).

The lung cancer cell panel was initially used to evaluate endogenous CYYR1 expression. A549 and H1299 cells, which exhibited very low or undetectable endogenous CYYR1 expression, were selected as the primary models for mechanistic studies, including protein interaction, ubiquitination, protein stability, and functional assays. Additional validation experiments were performed in H2122 and PC‐9 cells.

All cell lines were authenticated by short tandem repeat (STR) profiling before experimental use and were routinely tested for mycoplasma contamination. Only mycoplasma‐free cultures were used for subsequent experiments.

Different clinical specimen cohorts were used for different experimental purposes. A cohort of 21 paired LUAD tumor and adjacent normal tissues obtained from patients undergoing surgery at Chongqing University Cancer Hospital was used for qRT‐PCR and immunohistochemical validation of CYYR1 expression. An independent cohort of 192 LUAD tissues was used for promoter methylation analysis by MSP. Quantitative methylation analysis using the MassARRAY EpiTYPER platform was performed using 16 normal lung tissues and 56 LUAD tissues. In addition, circulating DNA methylation analysis was performed using cfDNA samples (*n* = 149), ctDNA samples (*n* = 136), and tDNA samples (*n* = 72). All patients provided written informed consent, and the study was approved by the Ethics Committee of Chongqing University Cancer Hospital (CZLS2022020‐A).

### 2.2 Kaplan–Meier Plotter

Kaplan–Meier Plotter analysis was performed to evaluate the association between CYYR1 expression and overall survival in LUAD patients. Patients were divided into high‐ and low‐expression groups according to the median expression level, and survival differences were evaluated using the log‐rank test.

### 2.3 Gene Expression and DNA Methylation Analysis

Total RNA was extracted from cells or tissues using TRIzol reagent (Thermo Fisher Scientific, 15596026) according to the manufacturer's protocol [20]. For reverse transcription‐PCR (RT‒PCR) and quantitative real‐time PCR (qPCR), 1 µg of total RNA was reverse‐transcribed into cDNA using the Prime Script RT Reagent Kit (Takara, RR047Q). PCR amplification was performed using specific primers (Table S1). For qPCR, reactions were carried out in triplicate using SYBR Green Premix (Roche, 4913850001) on a Quant Studio 6 Pro system (Applied Biosystems). The relative mRNA expression levels were calculated using the 2^–ΔΔCt^ method with β‐actin as the internal control.

DNA methylation analysis was performed using methylation‐specific PCR (MSP) and the MassARRAY EpiTYPER system (Agena Bioscience). Genomic DNA was treated with sodium bisulfite using the EZ DNA Methylation‐Gold Kit (Zymo Research, D5006). For MSP, bisulfite‐converted DNA was amplified with primers specific for methylated or unmethylated sequences of the CYYR1 promoter (Table S1). For the MassARRAY EpiTYPER analysis, a target region of the CYYR1 promoter was amplified by PCR using T7‐tagged primers. The resulting amplicons were subjected to in vitro transcription, RNase cleavage, and quantitative mass spectrometry to determine methylation levels at individual CpG sites. All reactions included negative (no template) and positive controls.

### 2.4 Gene Manipulation, Plasmid Construction, and Generation of Stable Cell Lines

Small interfering RNAs (siRNAs) targeting TRIM21 and negative control siRNAs were designed and synthesized by Gene Pharma (Shanghai, China; sequences provided in Table S2). For stable knockdown, two short hairpin RNAs (shRNAs) targeting HMMR (sh‐HMMR#1 and #2) were cloned into the pLV‐RNAi lentiviral vector (sequences in Table S2).

For overexpression studies in LUAD cell lines, the open reading frame (ORF) of CYYR1 was PCR‐amplified and cloned into the pCMV6‐entry vector (Ori Gene Technologies, PS100001) to generate the CYYR1‐OE plasmid. Stable CYYR1‐overexpressing cell lines were subsequently established through plasmid transfection and selection. Similarly, the ORF of HMMR was inserted into the pReceiver‐M07 vector (Genecopoeia) to construct the HMMR‐OE plasmid. The TRIM21 and CDC20 ORFs were individually subcloned into the pLV3‐CMV vector (Miao Ling Plasmid) to produce the TRIM21‐OE and CDC20‐OE plasmids, respectively. A pCMV‐HA vector (Clontech) was used to express ubiquitin (Ub). Transient transfections used Lipofectamine 3000. Stable cell lines were generated via lentiviral transduction as described [20, 21].

For epistasis and downstream bypass experiments, transient co‐transfection was performed to reintroduce downstream effectors (HMMR or CDC20) in CYYR1‐overexpressing cells or to restore CDC20 expression in HMMR‐depleted cells. Protein expression of the introduced constructs was confirmed by western blotting before subsequent functional assays. These transient epistasis/downstream‐bypass experiments were designed to evaluate pathway relationships and functional reversibility rather than to establish stable rescue cell models.

### 2.5 Cell Proliferation Assays

Cell viability was measured by CCK‐8 assay (BIOGROUND, BG0025). Cells (1,000/well) were seeded in 96‐well plates, and absorbance at 450 nm was recorded at indicated time points after CCK‐8 addition.

For colony formation, 1000 cells were seeded per well in 6‐well plates and cultured for 14 days. Colonies were fixed, stained with 0.1% crystal violet, and counted (≥ 50 cells/colony). All experiments were performed in triplicate.

### 2.6 Transwell Migration and Invasion Assays

Cell motility was assessed using Transwell chambers (8‐µm pore) (Corning, 3422). For invasion assays, chambers were pre‐coated with Matrigel (Corning, 356234). Serum‐starved cells (5 × 10^4^ for migration, 1 × 10^5^ for invasion) were seeded in the upper chamber, with 20% FBS‐containing medium used as a chemoattractant in the lower chamber. After incubation for a predetermined duration (24–48 h) based on preliminary optimization experiments and cellular migratory capacity, cells on the lower surface were fixed, stained, and counted. The same incubation duration was applied to all groups within each individual experiment.

### 2.7 Wound Healing Assay

Wound healing assays were performed to evaluate two‐dimensional cell migration. Cells were seeded in 6‐well plates and cultured to 90%–100% confluence. A sterile 200 µL pipette tip was used to generate a linear scratch wound. Detached cells were removed by washing with PBS, and the wounded monolayers were cultured in serum‐free medium. Images were acquired at 0 h and at a predefined observation time point (18 or 24 h) determined by preliminary optimization experiments. The observation duration was selected to ensure that wound closure remained within a measurable range and avoided complete closure or insufficient migration. The same observation time point was applied to all groups within each experiment.

### 2.8 Immunoprecipitation (IP) and Proteomics Analyses

Immunoprecipitation (IP) assays were performed as previously described with minor modifications [22, 23]. Briefly, cells were lysed in NP‐40 lysis buffer supplemented with protease inhibitor cocktail. After clarification by centrifugation, protein concentrations were determined, and equal amounts of total protein lysates were incubated with the indicated antibodies overnight at 4°C, followed by incubation with Protein A/G magnetic beads. After extensive washing, immunoprecipitated proteins were eluted for subsequent immunoblotting analysis or proteomic identification.

Different quantitative proteomic approaches were applied according to the specific biological questions addressed in this study. TMT‐based quantitative proteomics was used to characterize CYYR1‐associated proteomic changes and identify candidate downstream regulators, whereas label‐free quantitative proteomics was used to profile proteomic alterations following HMMR perturbation.

For CYYR1‐associated proteomic analyses, immunoprecipitated samples obtained from CYYR1‐associated protein screening and whole‐cell lysates from CYYR1‐overexpressing LUAD cells were subjected to reduction, alkylation, and tryptic digestion. Peptides were labeled with tandem mass tag (TMT) reagents according to the manufacturer's instructions. After labeling, peptides were fractionated by high‐pH reverse‐phase high‐performance liquid chromatography (HPLC) and analyzed by nano‐liquid chromatography tandem mass spectrometry (nano‐LC‐MS/MS) using an Orbitrap Exploris 480 mass spectrometer.

For HMMR perturbation‐associated proteomic analyses, whole‐cell lysates from HMMR‐manipulated LUAD cells were subjected to reduction, alkylation, and tryptic digestion, followed by label‐free quantitative proteomic analysis using nano‐LC‐MS/MS coupled with an Orbitrap Exploris 480 mass spectrometer.

Raw mass spectrometry data were processed and searched against the UniProt human protein database using Proteome Discoverer and Mascot search engines. For TMT‐based datasets, protein abundance was quantified based on reporter ion intensities, whereas label‐free quantitative datasets were quantified based on peptide intensity measurements. Protein identification was performed based on peptide‐spectrum matching. Proteins showing fold changes ≥ 1.5 and nominal *p* values < 0.05 were initially considered candidate proteins for exploratory prioritization. To account for multiple testing, Benjamini–Hochberg correction was additionally applied, and FDR‐adjusted *p* values are provided in the corresponding Supporting Tables. An FDR < 0.05 was considered statistically significant after multiple‐testing correction. Proteins meeting the initial fold‐change and nominal *p*‐value criteria but not the FDR threshold were retained only as hypothesis‐generating candidates and were not considered statistically significant after multiple‐testing correction. Because these proteomic datasets were designed as hypothesis‐generating discovery approaches rather than definitive standalone evidence of biological regulation, candidate proteins were further evaluated through integration with clinical datasets, computational analyses, and orthogonal experimental validation, including co‐immunoprecipitation, western blotting, ubiquitination assays, and functional epistasis analyses.

The processed quantitative proteomic datasets generated from CYYR1‐interacting protein screening, CYYR1‐overexpression TMT‐based quantitative proteomics, and HMMR perturbation label‐free quantitative proteomics are provided in Tables S4–S6.

### 2.9 Co‐Immunoprecipitation (Co‐IP) and Western Blot Analysis

Co‐immunoprecipitation (Co‐IP) assays were performed to investigate protein associations and ubiquitination events. Cells were lysed in NP‐40 buffer supplemented with protease inhibitors. For endogenous interaction analysis, approximately 500 µg of total protein lysates were incubated with the indicated antibodies overnight at 4°C, followed by incubation with Protein A/G Magnetic Beads (Enriching Biotechnology, P28). For epitope‐tagged protein interaction experiments, tag‐specific antibodies were used for immunoprecipitation. After extensive washing, immunoprecipitated proteins were eluted and subjected to SDS‐PAGE and Western blot analysis.

For epitope‐tagged protein interaction experiments, Myc‐tag immunoprecipitation was performed using Myc‐Tag (71D10) Rabbit Monoclonal Antibody (#2278, Cell Signaling Technology). HA‐tag immunoprecipitation was performed using HA Tag Polyclonal Antibody (51064‐2‐AP, Proteintech). His‐tag immunoprecipitation was performed using His‐tag polyclonal antibody (Rabbit) (10001‐0‐AP, Proteintech).

Proteins were separated by SDS‐PAGE and transferred onto PVDF membranes. Membranes were blocked with 5% BSA and incubated overnight at 4°C with primary antibodies. After incubation with HRP‐conjugated secondary antibodies, protein signals were visualized using enhanced chemiluminescence (ECL) substrate (Millipore).

The following antibodies were used for endogenous protein detection: anti‐HMMR (sc515221, Santa Cruz Biotechnology; 1:1000), anti‐TRIM21 (sc‐25351, Santa Cruz Biotechnology; 1:1000), anti‐CDC20 (sc‐5296, Santa Cruz Biotechnology; 1:1000), anti‐ubiquitin (sc‐8017, Santa Cruz Biotechnology; 1:1000), and anti‐β‐actin (sc‐47778, Santa Cruz Biotechnology; 1:3000).

For tagged protein detection, Myc‐tag western blotting was performed using Myc‐Tag (E4F4D) Mouse Monoclonal Antibody (#20229, Cell Signaling Technology; 1:2000). HA‐tag western blotting was performed using Recombinant Anti‐HA Tag antibody (Mouse mAb) (GB151252‐100, Servicebio). His‐tag western blotting was performed using Recombinant Anti‐His Tag antibody (Mouse mAb) (GB151251‐100, Servicebio).

Protein bands were visualized using enhanced chemiluminescence (ECL) substrate (Millipore) and imaged with a ChemiDoc MP Imaging System (Bio‐Rad). Representative immunoblot images from independent experiments are shown.

For ubiquitination assays, cells were co‐transfected with the indicated expression plasmids and HA‐ubiquitin constructs. Following immunoprecipitation of target proteins, ubiquitination levels were detected by immunoblotting with anti‐HA or anti‐ubiquitin antibodies. Where indicated, endogenous HMMR ubiquitination was assessed by immunoprecipitation of endogenous HMMR followed by immunoblotting with anti‐ubiquitin antibodies. TRIM21‐specific siRNAs were used to deplete TRIM21 expression prior to ubiquitination analysis where indicated. Please see Table S7 for more detailed antibody information.

### 2.10 Immunofluorescence Staining

Immunofluorescence (IF) experiments were performed as previously described [20, 23]. Cells were seeded on coverslips, fixed with 4% paraformaldehyde, permeabilized with 0.2% Triton X‐100, and blocked with 5% BSA.

Cells were incubated overnight at 4°C with primary antibodies against CYYR1 (HPA067685, Sigma–Aldrich; 1:200), TRIM21 (sc‐25351, Santa Cruz Biotechnology; 1:200), and HMMR (sc515221, Santa Cruz Biotechnology; 1:200).

After washing, cells were incubated with Alexa Fluor‐conjugated secondary antibodies for 1 h at room temperature in the dark. Nuclei were counterstained with DAPI (1 µg/mL; Sigma, D1306) for 5 min. Coverslips were mounted using ProLong Gold Antifade Mountant (Invitrogen).

For epitope‐tagged protein visualization, His‐tagged proteins were detected using Recombinant Anti‐His Tag antibody (Mouse mAb) (GB151251‐100, Servicebio).

Images were acquired using a Leica TCS SP8 confocal microscope. All experiments were independently repeated at least three times.

### 2.11 Animal Studies

All animal procedures were conducted in accordance with guidelines approved by the Animal Ethics Committee of Chongqing University Cancer Hospital (Approval No. CQCH‐LAE‐A0000202089) and performed in accordance with relevant guidelines. Female BALB/c nude mice (4‐6 weeks old) were housed under specific pathogen‐free (SPF) conditions.

To evaluate the role of CYYR1 and HMMR in tumor growth, A549 cells stably expressing the following constructs were used: control, CYYR1 overexpression, and HMMR knockdown. Cells were harvested and resuspended in a 1:1 mixture of PBS and Matrigel (BD Biosciences). A total of 5 × 10^6^ cells in 100 µL were injected subcutaneously into the bilateral flanks of each mouse (*n* = 5 biological replicates (individual mice)). Each mouse was considered as one biological replicate. Bilateral tumors generated from the same mouse were analyzed as paired measurements and were not considered independent experimental replicates. Tumor formation was palpable approximately 10 days post‐inoculation. Tumor length (L) and width (W) were measured every two days using a digital caliper, and volume was calculated as V = 0.5 × L × W^2^. Mice were euthanized when tumors reached a maximum diameter of 1.5 cm or at the study endpoint. Tumors were excised, weighed, photographed, and processed for further analysis.

For the orthotopic model, the same cell groups were prepared. Mice were anesthetized, and a small incision was made to expose the left lung. A suspension of 1 × 10^6^ cells in 50 µL of PBS mixed with 10% Matrigel was slowly injected into the left lung parenchyma. The wound was sutured, and mice were monitored until recovery. All mice were euthanized 30 days post‐injection. Lungs were harvested, and tumor nodules were counted and measured. Tissues were fixed for histopathological examination.

To assess lung colonization potential, 1 × 10^6^ cells in 100 µL PBS were injected into the lateral tail vein of mice (*n* = 5 biological replicates (individual mice)). Mice were monitored for signs of distress and were euthanized 30 days after tail‐vein injection. Lungs were collected, and surface metastatic foci were counted under a dissecting microscope. Tissues were then fixed for histological confirmation (H&E staining).

To model systemic dissemination, 1 × 10^6^ cells in 100 µL PBS were injected into the left ventricle of anesthetized mice. Metastatic progression was monitored every two weeks via in vivo bioluminescence imaging (BLI) using an IVIS Spectrum system (PerkinElmer) following D‐luciferin injection (150 mg/kg). At the experimental endpoint, major organs were harvested for ex vivo BLI and histopathological analysis.

### 2.12 In Vivo Imaging

For bioluminescence imaging experiments, LUAD cells were transduced with lentiviral vectors encoding firefly luciferase and selected to establish stable luciferase‐expressing cell populations. Luciferase activity was confirmed before animal implantation, and cells with comparable baseline luminescence were used for different experimental groups.

Mice were anesthetized with 2% isoflurane and injected intraperitoneally with 150 mg/kg D‐luciferin (Yeasen, 40901ES01) dissolved in PBS. After a 10‐min incubation period to allow for substrate distribution and peak signal emission, bioluminescent images were acquired using an IVIS Spectrum In Vivo Imaging System (Caliper Life Sciences, Alameda, CA, USA). Following image acquisition, data were quantified using Living Image Software 4.3.1 (Caliper Life Sciences). Regions of interest (ROIs) were drawn around the signal foci, and total flux (photons/second) was calculated for each ROI after subtracting background luminescence.

### 2.13 Immunohistochemistry (IHC) and Hematoxylin‐Eosin (H&E) Staining

Tumor tissues were fixed in 4% paraformaldehyde (HT501128; Sigma–Aldrich) for 24–48 h at room temperature, followed by paraffin embedding using standard histological procedures. Sections were cut at 4‐µm thickness using a rotary microtome (Leica, Wetzlar, Germany).

For H&E staining, sections were deparaffinized in xylene, rehydrated through a graded ethanol series, and stained with hematoxylin (GHS232; Sigma–Aldrich) for 5 min and eosin (HT110232; Sigma–Aldrich) for 2 min according to standard protocols. After dehydration and clearing, slides were mounted with neutral balsam (G8590; Solarbio).

For IHC staining, antigen retrieval was performed by heating sections in 10 mm sodium citrate buffer (pH 6.0) at 95°C for 20 min. After cooling and blocking endogenous peroxidase activity with 3% H_2_O_2_ for 15 min, sections were incubated with primary antibodies at 4°C overnight: anti‐CYYR1 (HPA067685, Sigma–Aldrich; 1:200), anti‐HMMR (sc515221, Santa Cruz Biotechnology; 1:200), anti‐CDC20 (sc5296, Santa Cruz Biotechnology; 1:200), and anti‐Ki67 (sc‐23900, Santa Cruz Biotechnology; 1:300), followed by HRP‐conjugated secondary antibodies and DAB development. IHC staining was semi‐quantitatively assessed by two independent pathologists blinded to sample information based on staining intensity and the proportion of positively stained cells according to established pathological criteria. Any discrepancies between the two observers were resolved by consensus.

### 2.14 Patient‐Derived Organoid Culture and Viability Assay

LUAD patient‐derived organoids were established from surgically resected tumor tissues as previously described [24], with minor modifications. Briefly, fresh tumor tissues were collected in Storage Medium (OneTar Biomedicine, PS‐100), minced and digested using Digestion Solution (OneTar Biomedicine, DB100) for 15–40 min at 37°C. The digested tissue was filtered through a 100‐µm cell strainer and embedded in Matrigel (Corning). Organoids were cultured in advanced DMEM/F12 medium supplemented with GlutaMax (Gibco, 21051024), HEPES (Gibco, 15630080), B27 (Gibco, 17504044), SB202190 (Selleck, S1077), Y‐27632 (Selleck, S6390), N‐acetylcysteine (Selleck, S1623), nicotinamide (Selleck, S1899), Noggin, R‐spondin‐1, FGF10, FGF7, and Wnt‐3a (all from PeproTech). The culture medium was refreshed every 2–3 days.

In this study, one independent LUAD patient‐derived organoid (PDO) line derived from one patient surgical tumor specimen was used for all organoid experiments. Organoids were expanded after stable growth was achieved and subsequently subjected to genetic manipulation and pharmacological treatment.

For CYYR1 restoration experiments, patient‐derived organoids were dissociated into single cells using TrypLE Express (Gibco) and transduced with a lentiviral vector carrying human CYYR1 (GenePharma, Shanghai, China). Lentiviral transduction was performed at an MOI of 20 in the presence of 8 µg/mL polybrene for 4 h. Empty‐vector lentivirus was used as the corresponding control. Successful CYYR1 expression restoration was confirmed by downstream molecular analyses.

For drug‐response experiments, PDOs were treated with Cisplatin (CDDP, 10 µm), Genistein (50 µm), or the combination of Genistein and CDDP under the indicated experimental conditions.

Organoid viability was assessed using the CellTiter‐Glo 3D Cell Viability Assay (Promega, G9681) according to the manufacturer's instructions. Briefly, organoids were dissociated into single cells, seeded in 384‐well ultralow‐attachment plates at a density of 2000 cells per well, and allowed to reform organoids for 4 days. Subsequently, CellTiter‐Glo 3D reagent was added, and luminescence was measured using a microplate reader. Relative viability was normalized to the control group. Each experimental condition was evaluated using three technical replicates.

### 2.15 Single‐Cell and Spatial Transcriptomic Analysis

To characterize the cellular distribution and spatial expression patterns of CYYR1, HMMR, and CDC20 within the NSCLC tumor ecosystem, publicly available single‐cell RNA sequencing and spatial transcriptomic datasets were analyzed. Because the available datasets include metastatic NSCLC specimens, these analyses were considered complementary evidence for gene expression patterns and cellular localization rather than direct evidence of LUAD‐specific molecular regulation.

The single‐cell transcriptomic dataset GSE223499 was obtained from the Gene Expression Omnibus (GEO) database [25]. Single‐cell RNA sequencing data processing was performed using the Seurat package. Briefly, low‐quality cells and potential doublets were removed according to standard quality control procedures. Data normalization and variance stabilization were performed using the SCTransform method. Principal component analysis (PCA) was subsequently conducted for dimensionality reduction, followed by uniform manifold approximation and projection (UMAP) visualization and graph‐based clustering. Major cell populations were annotated according to canonical lineage marker genes. The epithelial/tumor cell population was further extracted for downstream analysis. The expression patterns of CYYR1, HMMR, and CDC20 were compared between normal epithelial cells and malignant epithelial cells. Considering the sparsity of single‐cell transcriptomic data, expression distributions were visualized using violin plots with embedded box plots.

Spatial transcriptomic analysis was performed using the publicly available 10x Genomics Visium dataset GSE179572 (subsample GSM5420751) obtained from the GEO database [26]. This sample was derived from a patient with lung adenocarcinoma and was analyzed to evaluate the spatial localization and regional expression patterns of CYYR1, HMMR, and CDC20 within the LUAD tissue context.

The spatial transcriptomic analysis was based on the original 10x Visium Seurat object. Raw count matrices were imported and normalized using the Seurat Log Normalize method with a scale factor of 10 000. Spatial expression patterns of CYYR1, HMMR, and CDC20 were visualized using Seurat Spatial FeaturePlot, where each spatial spot represented a Visium capture region (approximately 55 µm in diameter and containing multiple cells). Both raw count‐based visualization (slot = “counts”) and normalized expression visualization (slot = “data”) were generated to evaluate absolute signal intensity and normalized regional expression differences, respectively.

To characterize tumor‐associated spatial regions, spatial deconvolution was performed using the Cottrazm framework (Construction of Tumor Transition Zone Microenvironment). Cottrazm reconstructs the spatial tumor microenvironment through three sequential steps: (1) Cottrazm‐Boundary Define integrates spatial transcriptomic profiles with corresponding H&E images to identify tumor boundary regions; (2) Cottrazm‐SpatialDecon integrates single‐cell RNA sequencing data, spatial transcriptomic data, and spatial coordinates to estimate the cellular composition of each spatial spot; and (3) Cottrazm‐SpatialRecon reconstructs cell‐type‐specific gene expression profiles at sub‐spot resolution [27, 28].

Based on the predicted proportion of malignant cells (Tumor Cells proportion) generated by Cottrazm‐Spatial Decon, spatial spots were classified into three categories: malignant regions (Tumor Cells = 1), normal regions (Tumor Cells = 0), and mixed malignant regions (0< Tumor Cells < 1). The average expression levels of CYYR1, HMMR, and CDC20 were calculated within each spatial category.

For statistical comparison among spatial regions, Wilcoxon rank‐sum tests were performed for pairwise comparisons between malignant, mixed malignant, and normal regions. Statistical results were presented together with group means, standard errors, and sample sizes. These spatial analyses were used to determine whether candidate genes preferentially localized to malignant cell‐enriched regions and provided complementary support for their tumor‐associated expression patterns.

### 2.16 Construction of a Prognostic Model Based on Multiple Machine‐Learning Algorithms

To construct a prognostic model for LUAD, candidate genes were derived from integrated multi‐cohort transcriptomic analyses. The TCGA‐LUAD cohort was used as the primary cohort for model development and internal evaluation. Patients were randomly divided into training and testing datasets at a 7:3 ratio, and additional GEO cohorts with available survival information were used for cross‐cohort evaluation of model performance.

Six survival modeling algorithms were evaluated, including Stepwise Cox regression, LASSO regression, Ridge regression, Elastic Net, Random Survival Forest (RSF), and CoxBoost. Different combinations of feature‐selection approaches and survival modeling algorithms were systematically evaluated, resulting in 113 candidate modeling configurations. Model performance was assessed using Harrell's concordance index (C‐index), calculated from internal training/testing datasets. When applicable, additional GEO cohorts with available survival information were used for further cross‐cohort evaluation of model generalizability.

Hyperparameters for penalized regression models were optimized through cross‐validation according to the corresponding algorithm framework. The machine‐learning framework was used for objective model prioritization and performance comparison rather than mechanistic inference.

### 2.17 Molecular Docking Analysis of Genistein With CDC20

The crystal structure of human CDC20 was obtained from the Protein Data Bank (PDB ID: 4GGC). The structure was prepared by removing water molecules, adding hydrogen atoms, and assigning partial charges using AutoDockTools. The chemical structure of Genistein was obtained in SDF format from PubChem and converted into PDB format using Open Babel. Both protein and ligand structures were further preprocessed, including hydrogen addition, charge assignment, and rotatable bond definition, using AutoDockTools (version 1.5.7).

Docking simulations were performed using AutoDock Vina (version 1.2.7) with an exhaustiveness parameter of 32. The docking procedure was used to predict potential binding conformations rather than to establish definitive direct binding. The pose with the lowest predicted binding free energy (ΔG) was selected for visualization and further structural analysis. Discovery Studio (version 2019) was used to generate two‐dimensional interaction diagrams illustrating predicted hydrogen bonds, hydrophobic contacts, and π‐π stacking interactions.

### 2.18 Cellular Thermal Shift Assay (CETSA)

A549 cells were treated with Genistein (50 µm) or vehicle control for 4 h and subsequently harvested for cellular thermal shift assay (CETSA). Cells were resuspended in phosphate‐buffered saline (PBS) supplemented with protease inhibitor cocktail and divided into aliquots. Cell suspensions were heated at indicated temperatures (37°C, 42°C, 47°C, 52°C, 57°C, and 62°C) for 3 min using a thermal cycler, followed by immediate cooling at room temperature. Cells were then subjected to repeated freeze–thaw cycles to facilitate cell lysis, and insoluble protein aggregates were removed by centrifugation. Equal amounts of soluble protein fractions were collected and analyzed by western blotting using an anti‐CDC20 antibody. GAPDH (Proteintech, 60004‐1‐Ig) was used as a loading control to assess sample consistency. The thermal stability of CDC20 was evaluated by comparing the remaining soluble CDC20 protein levels between Genistein‐treated and control groups across different temperatures.

### 2.19 Statistical Analysis

Statistical analyses were performed using GraphPad Prism 8.0 and R software. Statistical tests were selected according to data distribution and experimental design. For comparisons between two groups, two‐tailed Student's *t*‐test was used for normally distributed datasets, whereas the Mann–Whitney U test was applied for non‐normally distributed datasets. For comparisons involving multiple groups, one‐way analysis of variance (ANOVA) followed by Tukey's multiple‐comparison test was performed when applicable. Correlation analyses were conducted using Pearson or Spearman correlation coefficients according to data distribution.

For transcriptomic analyses, differential expression analyses were performed using appropriate multiple‐testing correction, and adjusted *p* values were used to determine statistical significance. Survival differences were evaluated using Kaplan–Meier analysis and Cox proportional hazards regression models, with hazard ratios (HRs) and 95% confidence intervals (CIs) reported where applicable.

For proteomic analyses, proteins meeting the predefined fold‐change and nominal *p*‐value criteria were initially retained for exploratory prioritization. Benjamini‐Hochberg correction was subsequently applied to account for multiple testing, and FDR‐adjusted *p* values are reported in the corresponding Supporting Tables. An FDR <0.05 was considered statistically significant after multiple‐testing correction, whereas candidates meeting only the initial nominal criteria were treated as hypothesis‐generating.

All in vitro experiments were independently repeated at least three times, and data are presented as mean ± standard deviation (SD) unless otherwise indicated. For animal experiments, each mouse represented one biological replicate, and sample sizes are indicated in the corresponding figure legends. Statistical significance was defined as *p* <0.05, or FDR <0.05 where multiple‐testing correction was applied.

## 3 Results

### 3.1 Integrative Multi‐Cohort Analysis and Machine Learning Identify CYYR1 as a Potential Tumor Suppressor and Prognostic Marker in LUAD

To identify highly robust prognostic biomarkers for LUAD, we performed differential expression analyses across the TCGA‐LUAD cohort and six independent GEO microarray cohorts (GSE118370, GSE140797, GSE10799, GSE40791, GSE130779, and GSE32863). The intersection of results via a Venn diagram nominated 14 commonly dysregulated candidate genes that were highly conserved in LUAD (Figure 1A and Table S3). Subsequently, we comprehensively evaluated 113 candidate prognostic modeling configurations generated by integrating multiple feature‐selection and survival modeling strategies (Figure 1B). To further validate the clinical relevance of these 14 genes, we calculated risk scores based on six classical machine learning algorithms (CoxBoost, Stepwise Cox, LASSO, Ridge, Elastic Net, and RSF). Using the TCGA‐LUAD cohort as the evaluation cohort, Kaplan–Meier survival analysis demonstrated that the 14‐gene signature stratified patients into high‐ and low‐risk groups with significantly different overall survival (Figure 1C). To investigate the specific contribution of each gene within the model, we extracted the iteration trajectory from the CoxBoost algorithm (Figure 1D) and a bar plot of feature coefficients (Figure 1E). The results revealed that CYYR1 represented the strongest candidate among the prioritized genes (protective gene) in the model, suggesting that CYYR1 may represent a candidate tumor‐suppressive regulator in LUAD. The ROC curve analysis demonstrated that CYYR1 expression showed strong discrimination between LUAD tumor and available normal samples within the TCGA dataset (AUC = 0.984) (Figure 1F).

**FIGURE 1:**
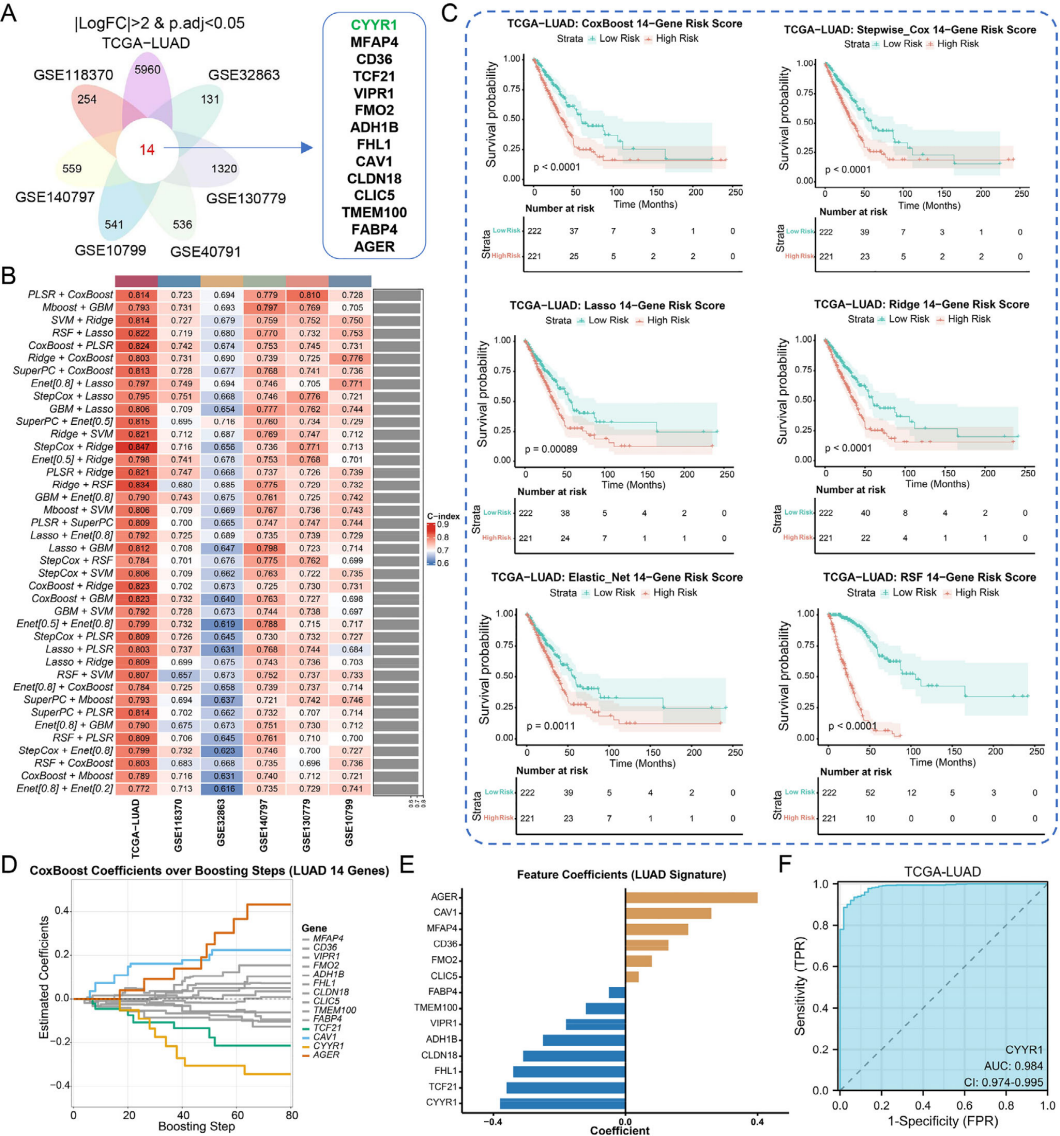
Integrative multi‐cohort analysis identifies CYYR1 as a candidate LUAD‐associated biomarker. (A) Venn diagram of differentially expressed genes (DEGs) from six integrated LUAD GEO datasets (GSE118370, GSE140797, GSE10799, GSE40791, GSE130779, GSE32863) and the TCGA‐LUAD dataset, identifying 14 commonly dysregulated genes. (B) Heatmap illustrating the cross‐cohort C‐index evaluation of 113 machine‐learning‐based survival model combinations. The heatmap displays the predictive performance of the top 40 models within the TCGA‐LUAD training cohort and across five GEO cohorts used for cross‐cohort performance evaluation. (C) Kaplan–Meier curves demonstrated a highly significant difference in overall survival (OS) between patients in the high‐risk group (red line) and the low‐risk group (blue line), stratified by risk scores calculated using six mainstream models (LASSO, Ridge, Elastic Net, CoxBoost, Stepwise Cox regression, and Random Survival Forest). (D,E) Iteration trajectory of the CoxBoost algorithm (D) and feature coefficient distribution of the selected prognostic model (E). (F) Receiver operating characteristic (ROC) curve demonstrating the diagnostic performance of CYYR1 expression in distinguishing LUAD tissues from normal tissues in the TCGA‐LUAD cohort (AUC = 0.984).

Subsequently, we found that CYYR1 was downregulated in a variety of tumors, including LUAD (Figure 2A). To examine CYYR1 expression at single‐cell resolution in a complementary NSCLC reference dataset, we performed dimensionality reduction and clustering of the GSE223499 single‐cell transcriptomic data (Figure S1A,B). The UMAP plot clearly delineated seven major cellular lineages within the tumor microenvironment (Figure 2B). We specifically extracted the epithelial/tumor cell subpopulation, and violin plots showed significantly lower CYYR1 expression in tumor epithelial cells than in their normal counterparts (Figure 2C,D). Consistently, spatial transcriptomic analysis of a LUAD specimen showed that CYYR1 expression differed across malignant, mixed malignant, and normal regions and was significantly lower in malignant regions than in non‐malignant regions (Figure S1C,D and Figure 2E,F). Validation in an independent cohort of 21 paired LUAD tumor and adjacent normal tissues confirmed that CYYR1 was significantly downregulated at both mRNA and protein levels (Figure 2G,H). Consistently, RT‐PCR analysis demonstrated very low or undetectable CYYR1 expression across the LUAD/NSCLC models selected for subsequent functional studies (Figure 2I). Prognostic analysis using the Kaplan–Meier Plotter indicated that higher CYYR1 expression was significantly associated with longer OS in LUAD patients (Figure 2J). Collectively, these findings demonstrate that CYYR1 is downregulated in LUAD and may contribute to LUAD progression.

**FIGURE 2:**
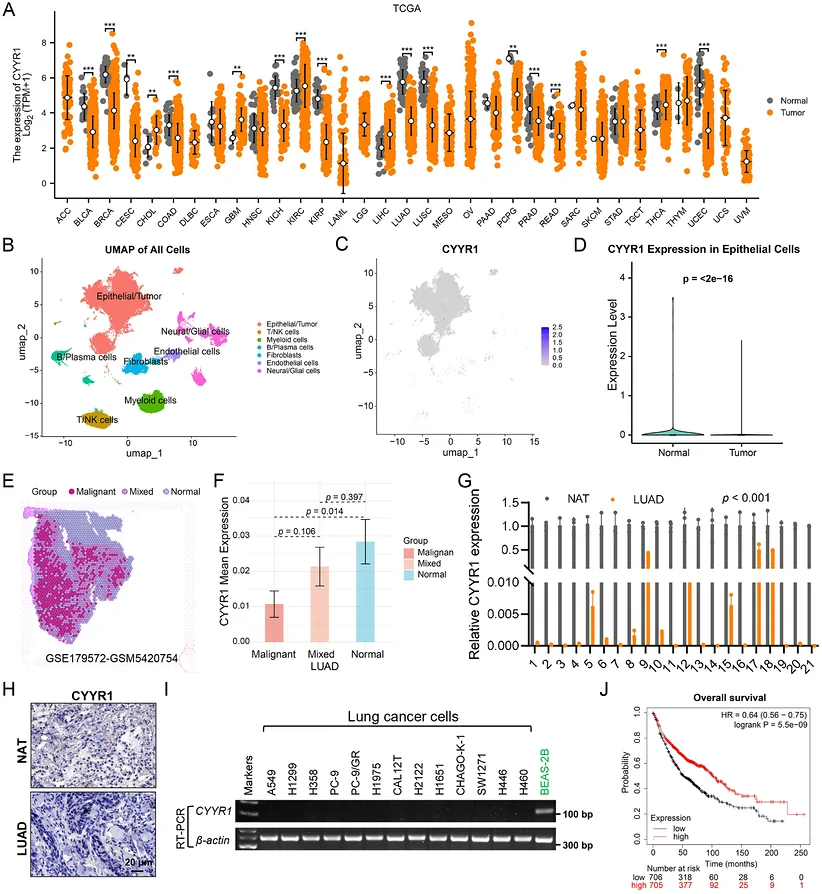
CYYR1 is downregulated in LUAD and associated with unfavorable clinical outcomes. (A) The expression of CYYR1 in pan‐cancer (data from TCGA). (B) UMAP visualization of annotated cell types. (C) UMAP visualization showing CYYR1 expression specifically within the epithelial cell cluster. (D) A violin plot depicting the expression of CYYR1 in normal epithelial cells vs. tumor epithelial cells. (E) Spatial features of malignant, mixed malignant, and normal regions within individual spots. (F) Quantification of CYYR1 expression across regions. (G) CYYR1 mRNA expression in 21 paired LUAD tumor and adjacent normal tissues measured by qRT‐PCR. (H) Representative immunohistochemical (IHC) images showing downregulation of CYYR1 protein in LUAD tissues compared to adjacent normal tissues. Scale bar, 20 µm. (I) CYYR1 mRNA expression in lung cancer cell lines, detected by RT‐PCR. (J) Kaplan–Meier survival curves showing the correlation between CYYR1 expression and overall survival in LUAD patients. ^*^ *p* < 0.05, ^**^ *p* < 0.01, ^***^ *p* < 0.001.

### 3.2 Promoter Hypermethylation Contributes to CYYR1 Downregulation in LUAD

Promoter hypermethylation represents a key mechanism of transcriptional regulation [29]. Analysis of the TCGA‐LUAD dataset revealed elevated methylation levels at the CYYR1 promoter region, which correlated inversely with CYYR1 expression (Figure 3A,B). MSP analysis demonstrated increased CYYR1 promoter methylation in lung cancer cell lines compared with normal lung epithelial cells (Figure 3C). To determine whether promoter methylation contributes to CYYR1 silencing, A549 and H1299 LUAD cells were treated with the DNA methyltransferase inhibitor 5‐Aza‐2′‐deoxycytidine (Aza) and the histone deacetylase inhibitor Trichostatin A (TSA). MSP analysis demonstrated that combined Aza+TSA treatment reduced CYYR1 promoter methylation levels and increased the proportion of unmethylated alleles (Figure 3D,E). Consistently, RT‐PCR analysis showed restoration of CYYR1 expression following demethylation treatment (Figure 3F), supporting a role for promoter hypermethylation in CYYR1 transcriptional repression.

**FIGURE 3:**
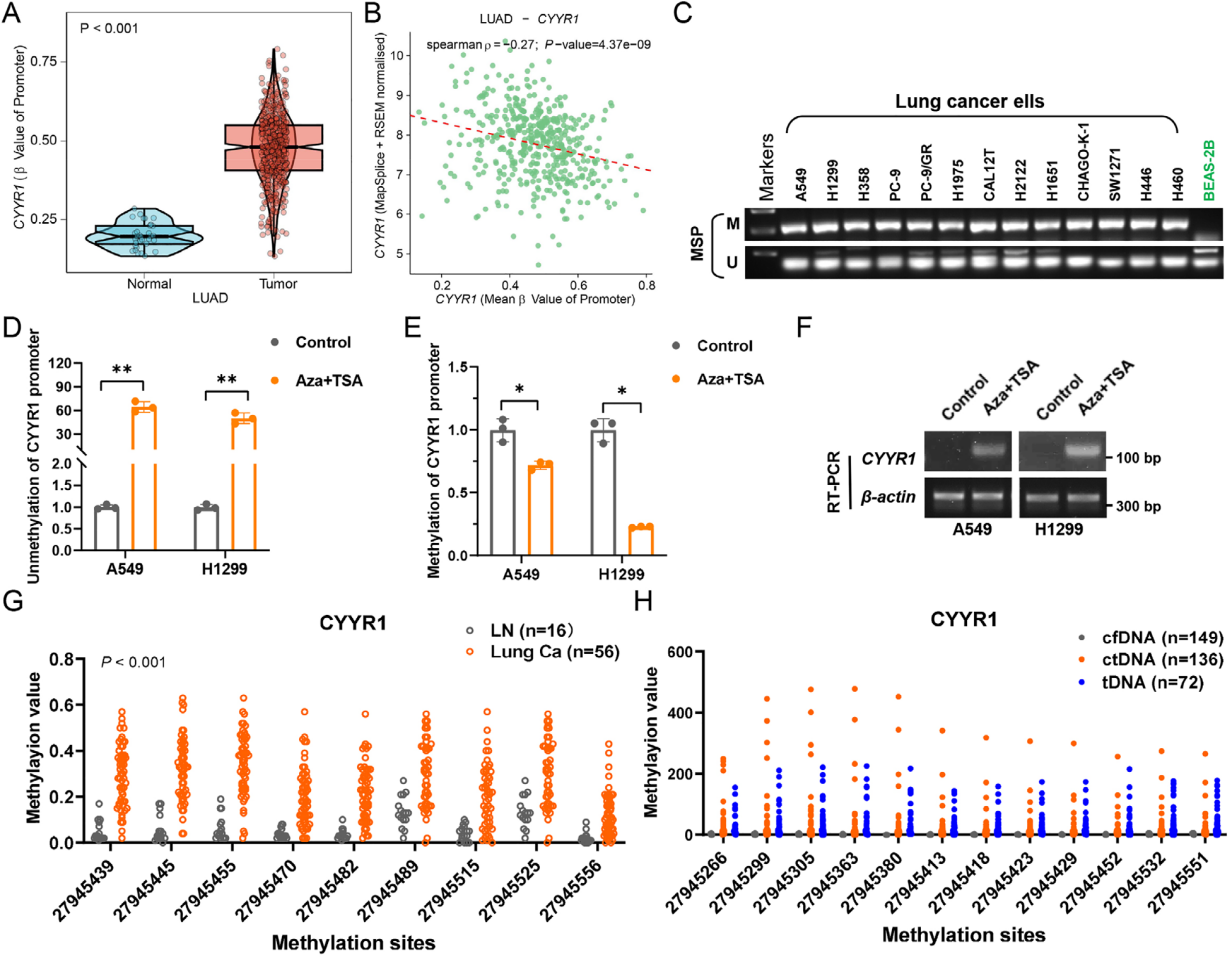
Promoter hypermethylation contributes to CYYR1 downregulation in LUAD. (A) Comparison of CYYR1 promoter methylation levels between LUAD and normal tissues from the TCGA‐LUAD cohort. (B) Correlation analysis between CYYR1 mRNA expression and promoter methylation levels in the TCGA‐LUAD cohort. (C) MSP analysis of CYYR1 promoter methylation status in lung cancer cell lines and normal lung epithelial cells. (D,E) Effect of DNA methyltransferase inhibition on CYYR1 promoter methylation in LUAD cells. Cells were treated with Aza and TSA, and promoter methylation changes were evaluated by MSP analysis. (F) Detection of CYYR1 expression restoration following Aza and TSA treatment in lung cancer cell lines by RT‐PCR. (G) Quantitative analysis of CYYR1 promoter methylation levels in normal lung tissues (*n* = 16) and LUAD tissues (*n* = 56) using the MassARRAY EpiTYPER platform. (H) Analysis of CYYR1 promoter methylation levels in cfDNA (*n* = 149), ctDNA (*n* = 136), and tDNA (*n* = 72) samples. ^*^ *p* < 0.05, ^**^ *p* < 0.01.

We next investigated the clinical relevance of CYYR1 promoter methylation. MSP analysis of an independent cohort of 192 LUAD tissues revealed frequent CYYR1 promoter methylation, with detectable methylation signals observed in 188 of 192 samples (Figure S3A). Bioinformatic prediction identified CpG islands within the CYYR1 promoter (Figure S3B). Quantitative methylation analysis using the MassARRAY EpiTYPER platform further confirmed increased CYYR1 promoter methylation levels in LUAD tissues compared with normal lung tissues using an independent cohort consisting of 16 normal lung tissues and 56 LUAD tissues (Figure 3G). Furthermore, analysis of circulating and tissue DNA specimens revealed CYYR1 promoter methylation alterations in cfDNA (*n* = 149), ctDNA (*n* = 136), and tDNA (*n* = 72) samples (Figure 3H).

### 3.3 CYYR1 Restoration Suppresses LUAD Tumor Growth and Metastatic Phenotypes In Vitro and In Vivo

To investigate the biological effects of restoring CYYR1 expression in CYYR1‐deficient LUAD cells, we stably overexpressed CYYR1 in A549 and H1299 cell lines (Figure 4A). CCK‐8 assays demonstrated that CYYR1 restoration significantly suppressed LUAD cell viability (Figure 4B). Colony formation assays further confirmed that restoration of CYYR1 reduced the proliferative capacity of both cell lines (Figure 4C). Flow cytometry analysis revealed increased G1‐phase accumulation following CYYR1 restoration in both A549 and H1299 cells (Figure 4D), suggesting impaired cell‐cycle progression associated with reduced proliferative capacity. To further determine whether the inhibitory effects of CYYR1 restoration were dependent on a specific cellular background, we extended our validation to additional LUAD cell lines, including H2122 and PC‐9 cells, which also exhibit low endogenous CYYR1 expression. Consistently, restoration of CYYR1 significantly reduced cell proliferation and clonogenic capacity in these additional models (Figure S4A–C), supporting the reproducibility of the inhibitory effects associated with CYYR1 restoration across multiple CYYR1‐deficient LUAD cellular contexts.

**FIGURE 4:**
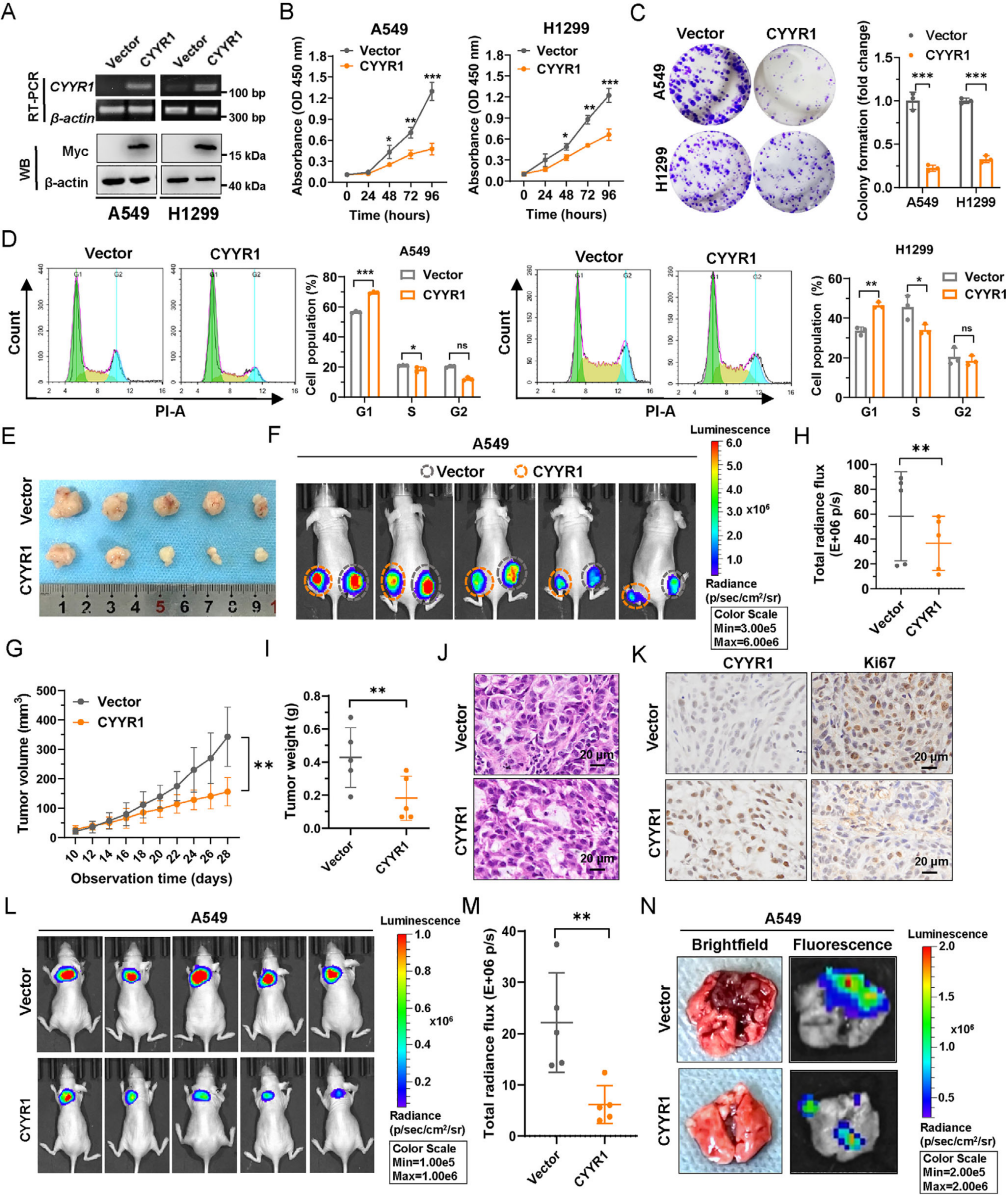
CYYR1 restoration suppresses LUAD tumor growth in vitro and in vivo. (A) Detection of CYYR1 overexpression efficiency by RT‐PCR and Western blot analysis in LUAD cells. (B, C) Proliferation of LUAD cells assessed by CCK‐8 assay (B) and clonogenic colony formation assay (C). (D) Flow cytometric analysis of cell cycle distribution in LUAD cells following CYYR1 overexpression. Effects of CYYR1 restoration on subcutaneous tumor growth in nude mice. (E) Representative images of excised subcutaneous tumors. (F) Bioluminescence imaging of subcutaneous tumors in nude mice. (G) Subcutaneous tumor growth curve. (H) Quantification of subcutaneous tumor bioluminescence signals. (I) Subcutaneous tumor weight. (J) Histological analysis of subcutaneous tumors by H&E staining. Scale bar, 20 µm. (K) Immunohistochemical (IHC) staining for CYYR1 and Ki67. Scale bar, 20 µm. (L) Bioluminescence imaging of orthotopic lung tumors in nude mice. (M) Quantification of orthotopic tumor bioluminescence signals. (N) Representative bright‐field and fluorescence images of orthotopic lung tumors. ^*^ *p* < 0.05, ^**^ *p* < 0.01, ^***^ *p* < 0.001.

We next examined the effect of CYYR1 restoration on tumor growth in vivo using subcutaneous xenograft models. Compared with the control group, tumors derived from CYYR1‐overexpressing A549 cells showed significantly reduced tumor growth, weight, and bioluminescence signal intensity (Figure 4E–I). Histological examination by H&E staining further demonstrated altered tumor morphology in CYYR1‐restored xenografts (Figure 4J), while IHC analysis showed reduced expression of the proliferation marker Ki67 (Figure 4K). In an orthotopic lung tumor model, mice injected with CYYR1‐overexpressing cells exhibited significantly lower bioluminescence signals than those in the control group (Figure 4L–N).

We also evaluated the effects of CYYR1 restoration on LUAD metastatic phenotypes. Transwell migration and scratch‐wound healing assays revealed that CYYR1 overexpression impaired the migratory and invasive abilities of A549 and H1299 cells in vitro (Figure 5A–C). To further evaluate whether the inhibitory effects of CYYR1 restoration on metastatic phenotypes were reproducible across independent LUAD models, migration and invasion assays were additionally performed in H2122 and PC‐9 cells. Similar to the observations in A549 and H1299 cells, CYYR1 restoration significantly reduced migratory and invasive capacities in both cell lines (Figure S4D–F), indicating that these inhibitory effects were not restricted to A549 and H1299 cells. In a tail vein injection model, CYYR1 overexpression led to a notable reduction in bioluminescence signals in the lungs (Figure 5D,E). Consistently, ex vivo lung imaging and H&E examination supported reduced pulmonary metastatic involvement in the CYYR1‐restored group (Figure 5F,G). Furthermore, in the intracardiac injection model, CYYR1 restoration markedly reduced systemic metastatic dissemination, including bioluminescence signals associated with brain and bone metastatic sites (Figure 5H,I). Together, these results demonstrate that restoration of CYYR1 suppresses tumor growth and metastatic phenotypes across the CYYR1‐deficient LUAD models examined.

**FIGURE 5:**
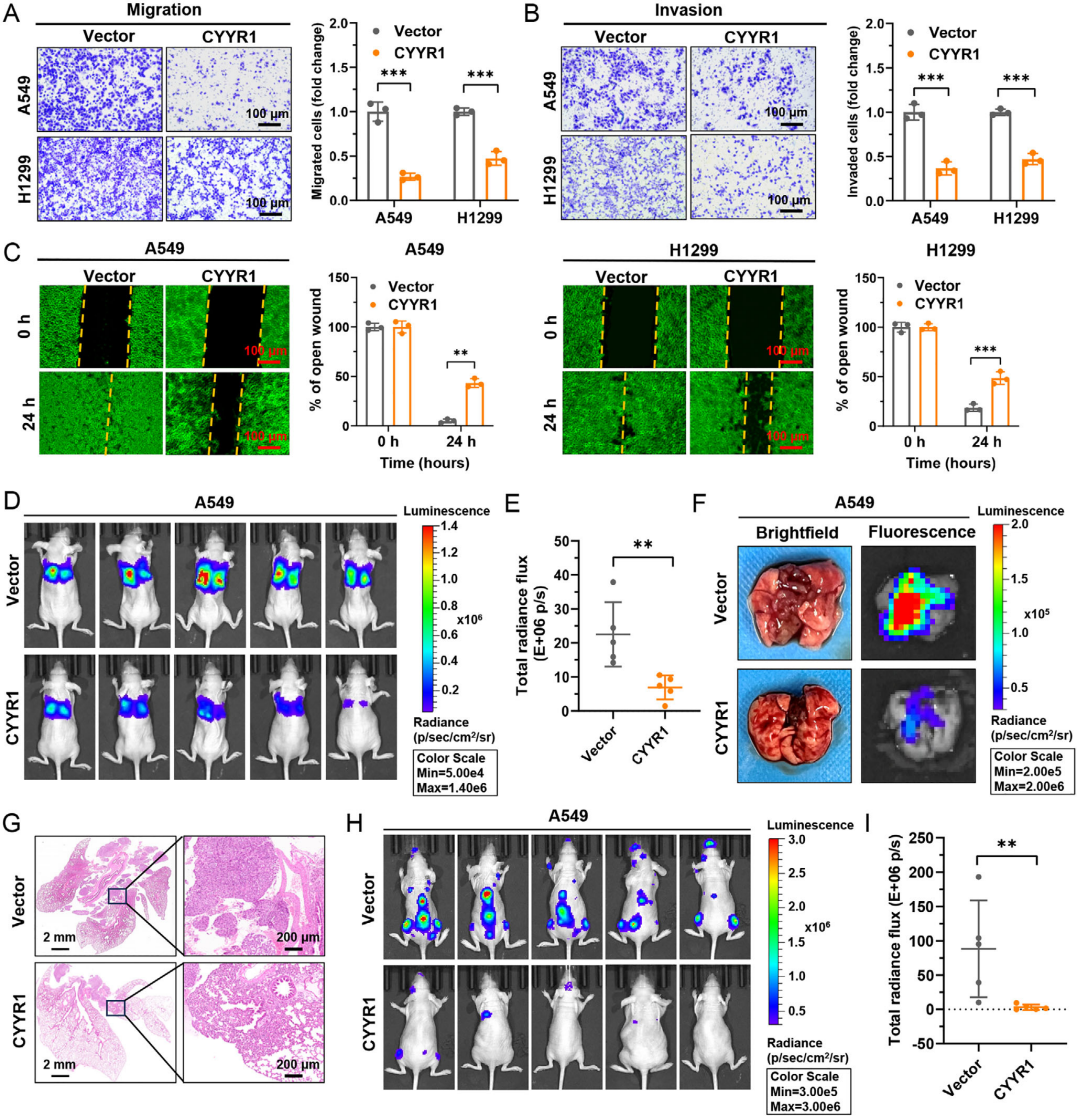
CYYR1 restoration suppresses LUAD metastatic phenotypes in vitro and in vivo. (A, B) Transwell assays evaluating the effects of CYYR1 overexpression on LUAD cell migration (A) and invasion (B) abilities. Scale bar, 100 µm. (C) Wound‐healing assay in A549 and H1299 cells stably expressing CYYR1. Images were taken at indicated time points. Scale bar, 100 µm. (D) In vivo bioluminescence imaging of metastatic dissemination 30 days after tail‐vein injection of luciferase‐labeled A549 cells. (E) Quantification of whole‐body bioluminescence signals. (F) Representative bright‐field and bioluminescence images of lungs from the tail‐vein injection model. (G) Lung tissue sections analyzed by hematoxylin and eosin (H&E) staining. Scale bar, 200 µm. (H) In vivo bioluminescence imaging of metastatic colonization 30 days after intracardiac injection of luciferase‐labeled A549 cells. (I) Quantification of bioluminescence signals from the intracardiac model. ^**^ *p* <0.01, ^***^ *p* <0.001.

### 3.4 CYYR1 Regulates HMMR Protein Stability Through a Proteasome‐Associated Mechanism in LUAD

To identify downstream effectors of CYYR1, we performed proteomic screening for its interacting partners in LUAD cells, identifying 83 candidate proteins enriched in processes such as protein transport and mismatch repair (Table S4, Figure S5A–C). Parallel TMT‐based quantitative proteomic analysis of CYYR1‐overexpressing cells identified 1084 candidate differentially abundant proteins according to the initial exploratory fold‐change and nominal *p*‐value criteria (Table S5, Figure S5D). Benjamini‐Hochberg correction was subsequently applied to account for multiple testing. Notably, HMMR, which was subsequently prioritized for mechanistic investigation, remained statistically significant after multiple‐testing correction (FDR < 0.05), and CDC20 also met the FDR < 0.05 threshold in the CYYR1‐overexpression TMT dataset (Table S5). KEGG and GO enrichment analyses of the CYYR1‐associated proteomic changes further highlighted cancer‐related pathways, including metabolism and RNA transport (Figure S5E,F).

Intersection of the interactome and proteome datasets identified six proteins that both bound to CYYR1 and were downregulated upon its overexpression (Figure 6A). To prioritize among these candidates, we applied ten distinct machine learning algorithms, which consistently prioritized HMMR for subsequent experimental investigation in LUAD pathogenesis (Figure 6B and Figure S6A,B). Clinical validation by immunohistochemistry, together with single‐cell transcriptomic analysis and spatial transcriptomic analysis of a LUAD specimen, further supported increased HMMR expression in tumor tissues and malignant epithelial compartments (Figure 6C and Figure S6C–E). Co‐IP assays supported an association between CYYR1 and HMMR under ectopic‐expression conditions, while western blotting showed that CYYR1 overexpression reduced HMMR protein abundance (Figure 6D,E). Immunofluorescence demonstrated their co‐localization and HMMR downregulation in CYYR1‐OE cells (Figure 6F).

**FIGURE 6:**
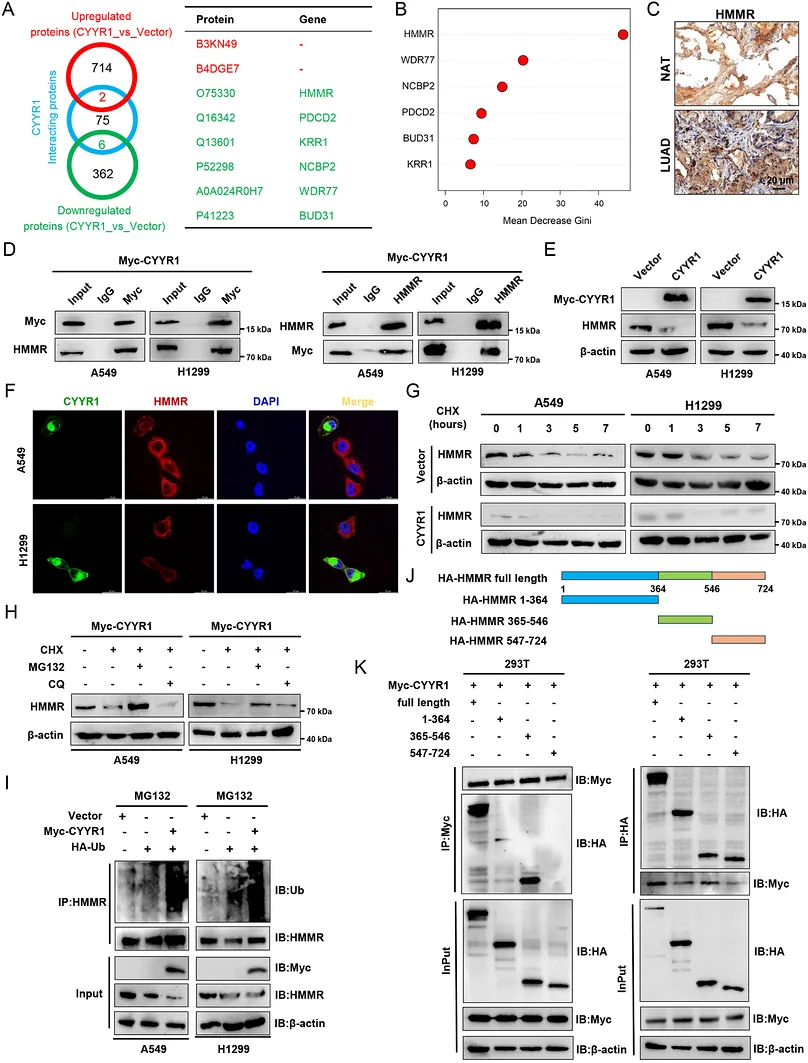
CYYR1 regulates HMMR protein stability in LUAD. (A) Quantitative proteomic analysis and Venn diagram of CYYR1‐interacting proteins. (B) Feature importance plot. The *x*‐axis denotes different features or genes; the *y*‐axis represents the Mean Decrease Gini score. (C) Representative immunohistochemical (IHC) images showing elevated HMMR expression in LUAD tissues compared to matched adjacent normal tissues. Scale bar, 20 µm. (D) Co‐immunoprecipitation (co‐IP) assay in A549 and H1299 cell lysates using anti‐Myc or anti‐HMMR antibodies, followed by immunoblotting. (E) Western blot analysis of HMMR protein levels following CYYR1 overexpression. (F) Immunofluorescence co‐staining for CYYR1 (green) and HMMR (red) in A549 and H1299 cells transfected with a CYYR1 plasmid. Nuclei were stained with DAPI (blue). Images were acquired by confocal microscopy. (G) HMMR protein stability assay. A549 and H1299 cells overexpressing CYYR1 were treated with cycloheximide (CHX) for the indicated times, followed by Western blot analysis. (H) Western blot analysis of HMMR expression in LUAD cells overexpressing CYYR1 and treated for 7 h with CHX, the proteasome inhibitor MG132, or the autophagy inhibitor chloroquine (CQ). (I) Ubiquitination assay. A549 and H1299 cells were co‐transfected with CYYR1 and HA‐ubiquitin plasmids for 48 h. HMMR was immunoprecipitated, and its ubiquitination status was assessed by immunoblotting with an anti‐HA antibody. (J) Schematic of HMMR truncation mutants. (K) Co‐IP assays in 293T cells co‐transfected with HA‐tagged HMMR truncation mutants and Myc‐tagged CYYR1 plasmids for 48 h. Immunoprecipitation was performed using anti‐HA or anti‐Myc antibodies, followed by immunoblotting to determine the interaction between CYYR1 and different HMMR regions. The HMMR 365‐546 region showed preferential association with CYYR1. All immunoblotting, co‐IP, ubiquitination, and immunofluorescence experiments shown were independently repeated three times, and representative images are presented.

Mechanistically, cycloheximide chase assays showed that CYYR1 overexpression reduced HMMR protein stability (Figure 6G). Treatment with MG132 partially restored HMMR protein abundance in CYYR1‐overexpressing cells, suggesting that proteasome‐associated degradation contributes to CYYR1‐mediated regulation of HMMR stability (Figure 6H). Consistently, ubiquitination assays revealed increased HMMR‐associated ubiquitination following CYYR1 overexpression (Figure 6I), indicating enhanced ubiquitination‐dependent regulation of HMMR. Domain‐mapping analysis further showed that CYYR1 preferentially associated with the HMMR 365‐546 region, identifying this region as a preferential CYYR1‐associated interface rather than establishing it as an exclusive functional domain for HMMR regulation (Figure 6J,K).

### 3.5 CYYR1 Facilitates TRIM21‐Dependent Ubiquitination and Degradation of HMMR in LUAD

Given that CYYR1 reduced HMMR protein stability through a proteasome‐associated mechanism, we next investigated whether an E3 ubiquitin ligase was involved in CYYR1‐mediated regulation of HMMR. Since CYYR1 lacks intrinsic ubiquitin ligase activity, we hypothesized that CYYR1 may facilitate E3 ligase‐dependent ubiquitination of HMMR. Co‐immunoprecipitation (co‐IP) assays demonstrated that CYYR1 associated with both HMMR and TRIM21 in CYYR1‐overexpressing A549 and H1299 cells (Figure 7A–C). Reciprocal immunoprecipitation further supported these protein associations under ectopic CYYR1‐expression conditions. Notably, CYYR1 overexpression did not significantly alter TRIM21 protein abundance, as determined by western blotting and immunofluorescence analysis (Figure 7D,E), suggesting that CYYR1 regulates TRIM21‐associated activity rather than TRIM21 expression itself. Immunofluorescence staining further revealed cytoplasmic co‐localization of CYYR1 and TRIM21 in LUAD cells (Figure 7E). Given that TRIM21 and HMMR are predominantly intracellular proteins, these observations suggest that the intracellular region of CYYR1 may contribute to their association, although the precise topology and assembly site of this regulatory complex require further investigation. However, because these interaction analyses relied predominantly on ectopic CYYR1 expression, the current data do not establish the existence, stoichiometry, or precise spatial organization of a physiological endogenous CYYR1‐TRIM21‐HMMR ternary complex. The endogenous interaction architecture and subcellular assembly site of this regulatory machinery therefore remain to be determined.

**FIGURE 7:**
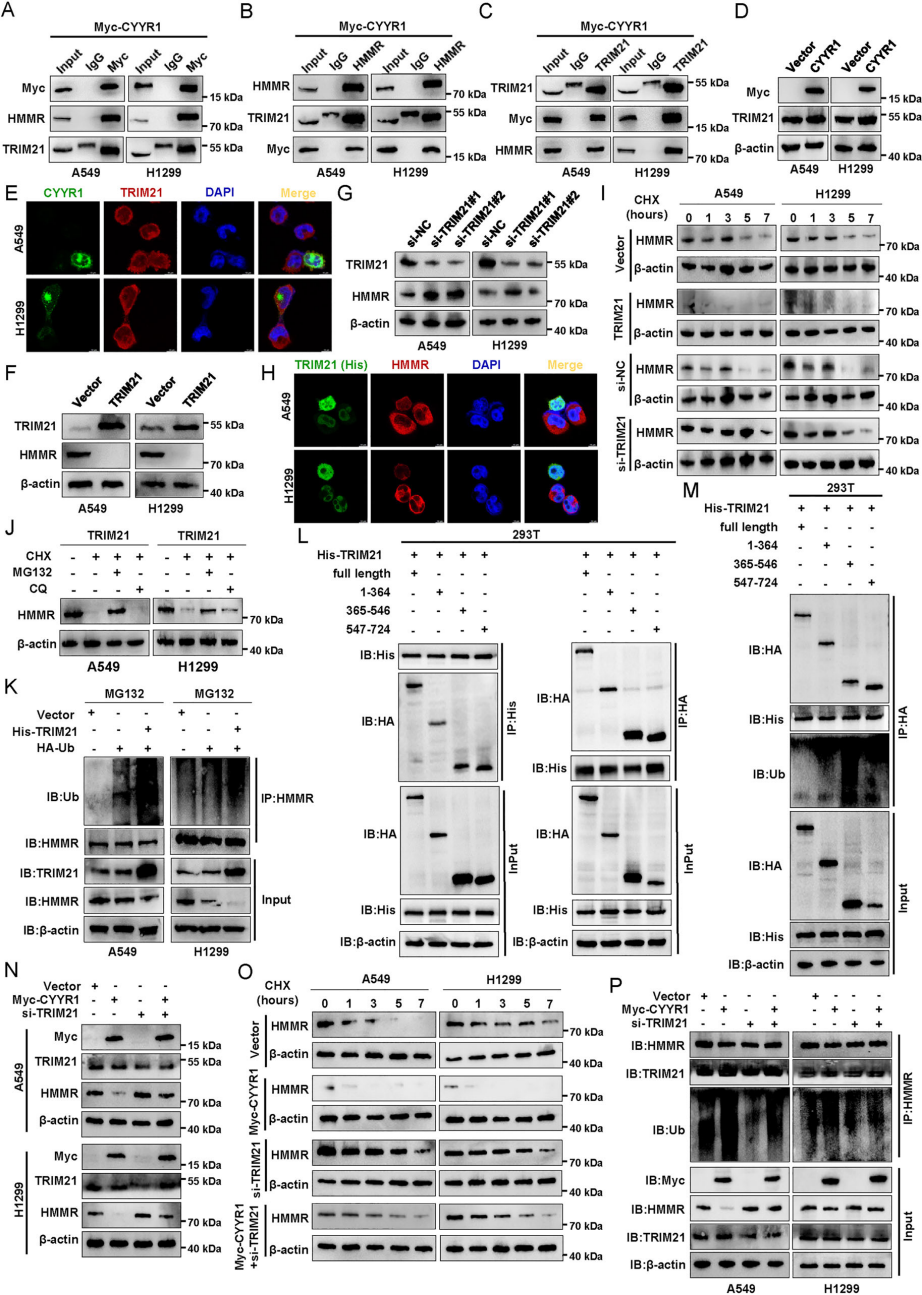
CYYR1 facilitates TRIM21‐dependent ubiquitination and degradation of HMMR in LUAD. (A–C) Co‐immunoprecipitation (co‐IP) assays supporting associations among CYYR1, HMMR, and TRIM21 in A549 and H1299 cells under ectopic CYYR1‐expression conditions. Cell lysates were immunoprecipitated using anti‐Myc (A), anti‐HMMR (B), or anti‐TRIM21 (C) antibodies, followed by immunoblotting with the indicated antibodies. (D) Western blot analysis showing that CYYR1 overexpression does not significantly alter TRIM21 protein abundance in A549 and H1299 cells. (E) Immunofluorescence staining showing the cytoplasmic co‐localization of CYYR1 (green) and TRIM21 (red) in A549 and H1299 cells following CYYR1 overexpression. Nuclei were counterstained with DAPI (blue). Images were acquired by confocal microscopy. (F,G) Western blot analysis of HMMR expression in A549 and H1299 cells following TRIM21 overexpression (F) or siRNA‐mediated TRIM21 knockdown (G). (H) Immunofluorescence co‐staining of His‐tagged TRIM21 (green) and endogenous HMMR (red) in A549 and H1299 cells transfected with a His‐TRIM21 plasmid. Nuclei were stained with DAPI (blue). Scale bar, 20 µm. (I) Cycloheximide (CHX)‐chase assays evaluating HMMR protein stability in A549 and H1299 cells following TRIM21 overexpression or knockdown. Cells were treated with CHX for the indicated time points and analyzed by western blotting. (J) Western blot analysis of HMMR protein levels in TRIM21‐overexpressing LUAD cells treated with CHX in combination with the proteasome inhibitor MG132 or the autophagy inhibitor chloroquine (CQ) for 7 h. (K) Ubiquitination assay showing TRIM21‐mediated ubiquitination of HMMR. A549 and H1299 cells were co‐transfected with TRIM21 and HA‐ubiquitin plasmids for 48 h. HMMR was immunoprecipitated, and ubiquitination levels were detected by immunoblotting with an anti‐HA antibody. (L) Schematic representation of HMMR truncation mutants used for interaction and ubiquitination analyses. (M) Domain mapping and ubiquitination analysis of HMMR truncation mutants. 293T cells were co‐transfected with HA‐tagged HMMR truncation constructs and His‐tagged TRIM21 plasmids. HMMR fragments were immunoprecipitated using anti‐HA antibodies, followed by immunoblotting with the indicated antibodies. TRIM21‐associated ubiquitination signals were detected across different HMMR regions, with relatively stronger ubiquitination observed in the 365‐546 fragment. These data do not establish this region as the exclusive or obligatory ubiquitination domain. (N) Western blot analysis showing that siRNA‐mediated TRIM21 depletion attenuates the CYYR1 overexpression‐induced reduction of HMMR protein abundance in A549 and H1299 cells. (O) CHX‐chase assays demonstrating that TRIM21 knockdown attenuates the accelerated HMMR degradation associated with CYYR1 restoration in A549 and H1299 cells. (P) HMMR ubiquitination assays showing that TRIM21 depletion attenuates CYYR1‐induced HMMR ubiquitination. HMMR was immunoprecipitated from A549 and H1299 cells under indicated conditions, followed by immunoblotting with an anti‐ubiquitin antibody. All co‐IP, western blotting, CHX‐chase, ubiquitination, and immunofluorescence experiments shown are representative of three independent experiments.

We next investigated whether TRIM21 regulates HMMR protein stability. Overexpression of TRIM21 markedly reduced HMMR protein levels in both A549 and H1299 cells (Figure 7F). Consistently, immunofluorescence analysis demonstrated decreased HMMR abundance and increased co‐localization between TRIM21 and HMMR following TRIM21 overexpression (Figure 7H). Cycloheximide (CHX) chase assays further revealed that TRIM21 overexpression accelerated HMMR degradation, whereas TRIM21 depletion by siRNA prolonged HMMR protein stability (Figure 7I). These findings indicate that TRIM21 negatively regulates HMMR protein stability in LUAD cells.

To determine the degradation pathway involved, LUAD cells overexpressing TRIM21 were treated with cycloheximide combined with either the proteasome inhibitor MG132 or the autophagy inhibitor chloroquine (CQ). MG132 treatment substantially restored HMMR protein abundance, whereas CQ treatment showed limited rescue effects (Figure 7J), suggesting that TRIM21 promotes proteasome‐associated degradation of HMMR.

We further examined whether TRIM21 regulates HMMR ubiquitination. Ubiquitination assays showed that TRIM21 overexpression significantly increased HMMR‐associated ubiquitination in A549 and H1299 cells (Figure 7K). Domain mapping experiments using HMMR truncation mutants revealed that TRIM21 associated with multiple regions of HMMR, including fragments spanning residues 1‐364, 365‐546, and 547‐724 (Figure 7L). Ubiquitination assays using HMMR truncation constructs revealed ubiquitination signals across multiple HMMR regions, with relatively stronger signals observed in the 365‐546 fragment (Figure 7M). Together with the preferential association of CYYR1 with HMMR residues 365‐546, these findings suggest that this region may represent a favored interface for CYYR1‐associated substrate positioning or accessibility during TRIM21‐dependent regulation. However, the present data do not establish that residues 365‐546 constitute the exclusive TRIM21‐binding region or an obligatory domain for HMMR ubiquitination and degradation. Bioinformatic analysis further indicated that potential ubiquitination sites are distributed across different regions of HMMR, supporting the possibility that TRIM21‐mediated ubiquitination may involve multiple accessible lysine residues and interaction surfaces rather than a single dominant ubiquitination site.

To determine whether TRIM21 is functionally required for CYYR1‐mediated regulation of HMMR, we performed genetic epistasis experiments by combining CYYR1 overexpression with TRIM21 depletion. Although CYYR1 overexpression markedly reduced HMMR protein abundance, this effect was substantially attenuated following TRIM21 knockdown in both A549 and H1299 cells (Figure 7N). Consistently, CHX chase assays demonstrated that TRIM21 depletion markedly reduced the accelerated HMMR degradation induced by CYYR1 restoration (Figure 7O). Furthermore, HMMR ubiquitination assays revealed that TRIM21 knockdown impaired the increase in HMMR‐associated ubiquitination triggered by CYYR1 overexpression (Figure 7P). Together, these findings demonstrate that TRIM21 acts as a critical mediator of CYYR1‐associated HMMR regulation and support a model in which CYYR1 facilitates TRIM21‐dependent ubiquitination and proteasomal degradation of HMMR in LUAD cells (Figure S7A,B). These functional dependency data support a CYYR1‐TRIM21‐HMMR regulatory relationship but do not by themselves establish the precise physiological architecture of an endogenous ternary complex.

To further determine whether TRIM21 is functionally required for CYYR1‐mediated tumor suppression, we performed functional epistasis experiments by combining CYYR1 overexpression with siRNA‐mediated TRIM21 depletion in A549 and H1299 cells. CYYR1 restoration significantly suppressed cell viability and colony‐forming ability, whereas TRIM21 knockdown partially reversed these inhibitory effects (Figure S8A,B). Similarly, TRIM21 depletion attenuated the suppressive effects of CYYR1 overexpression on cell migration and invasion, as demonstrated by Transwell migration/invasion assays and wound healing assays (Figure S8C–E). These findings further establish TRIM21 as a functional mediator of CYYR1‐induced LUAD suppression.

### 3.6 HMMR Promotes LUAD Proliferation and Metastasis In Vitro and In Vivo

Although HMMR has been reported as a diagnostic/prognostic marker in LUAD and has been implicated in NSCLC metastatic progression, its functional contribution and upstream protein‐stability regulation in LUAD remain incompletely characterized [19]. To investigate its oncogenic potential, we established stable HMMR‐knockdown LUAD cell lines through lentivirus‐mediated RNA interference (Figure 8A). Functional assays demonstrated that HMMR depletion significantly impaired malignant phenotypes in vitro: CCK‐8 assays revealed markedly reduced proliferation rates (Figure S9A), colony formation assays showed diminished clonogenic capacity (Figure S9B), and cell cycle analysis indicated increased G1‐phase accumulation in both A549 and H1299 cells (Figure S9C).

**FIGURE 8:**
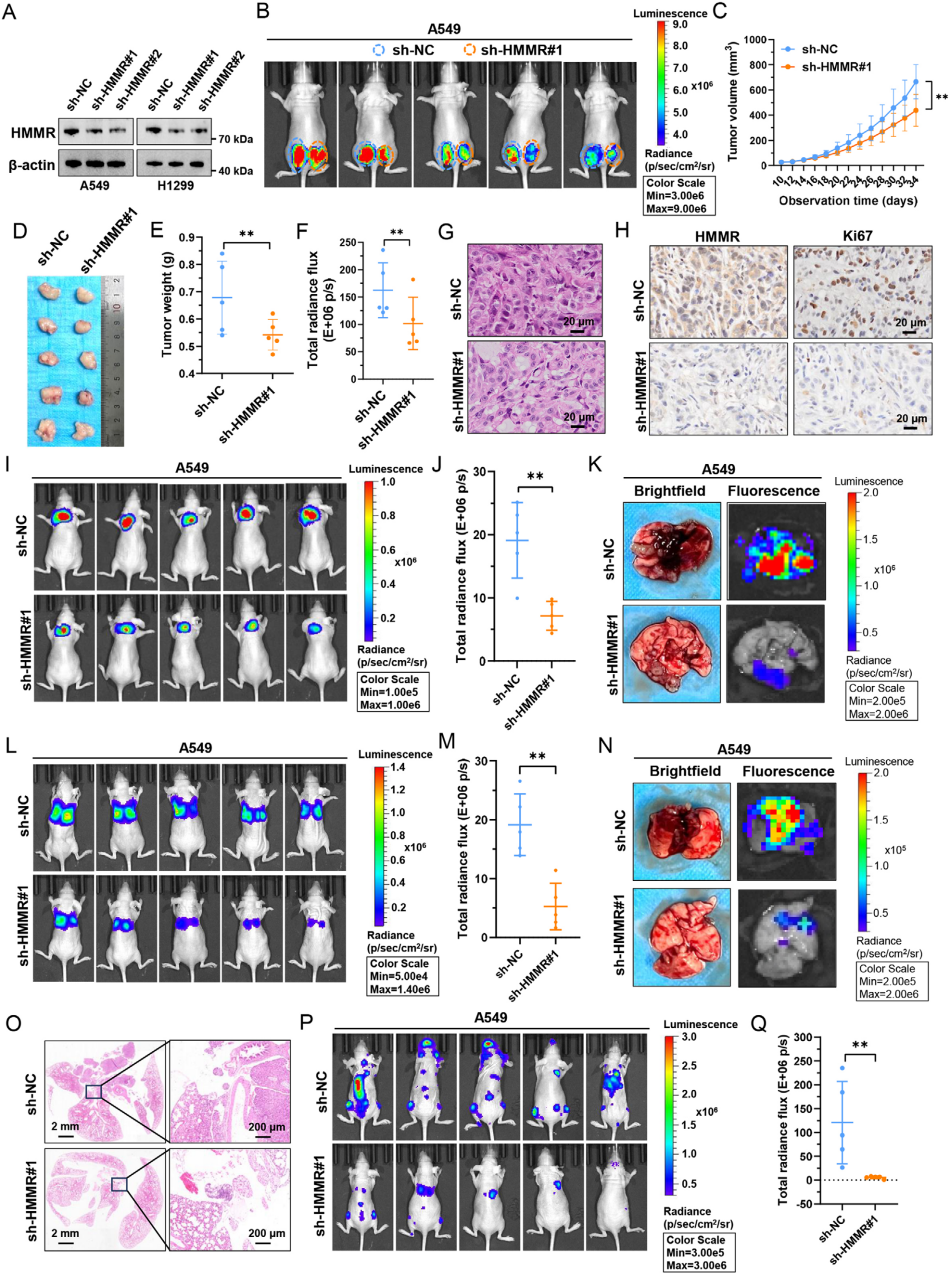
HMMR depletion suppresses LUAD tumor growth and metastatic dissemination in vivo. (A) Western blot analysis confirming HMMR knockdown efficiency in A549 cells. (B) Bioluminescence imaging of subcutaneous xenograft tumors in nude mice. (C) Quantification of subcutaneous tumor growth over time. (D) Representative images of excised subcutaneous tumors. (E) Quantification of tumor weight. (F) Quantification of bioluminescence signals from subcutaneous xenograft tumors. (G) Histological analysis of subcutaneous xenograft tumors by hematoxylin and eosin (H&E) staining. Scale bar, 20 µm. (H) Immunohistochemical staining for HMMR and Ki67 in subcutaneous xenograft tumors. Scale bar, 20 µm. (I) In vivo bioluminescence imaging of orthotopic lung tumors. (J) Quantification of orthotopic tumor bioluminescence signals. (K) Representative bright‐field and bioluminescence images of orthotopic lung tumors. (L) In vivo bioluminescence imaging following tail‐vein injection of luciferase‐labeled A549 cells. (M) Quantification of bioluminescence signals from the tail‐vein injection model. (N) Representative bright‐field and bioluminescence images of lungs from the tail‐vein injection model. (O) Histological analysis of lung tissues by H&E staining. Scale bar, 200 µm. (P) In vivo bioluminescence imaging following intracardiac injection of luciferase‐labeled A549 cells. (Q) Quantification of bioluminescence signals from the intracardiac injection model. ^*^ *p* < 0.05, ^**^ *p* < 0.01, ^***^ *p* < 0.001.

We next validated these findings using multiple in vivo models. In subcutaneous xenograft models, HMMR knockdown resulted in substantially reduced tumor volume, weight, and bioluminescence signals compared to control groups (Figure 8B–F). Histopathological examination further demonstrated decreased cellular density (Figure 8G) and reduced Ki67‐positive cell populations (Figure 8H) in HMMR‐deficient tumors. Through orthotopic lung implantation models, we confirmed that HMMR promotes primary tumor growth within the native lung microenvironment (Figure 8I–K).

The role of HMMR in metastatic progression was subsequently investigated. Transwell migration and scratch wound healing assays consistently showed that HMMR inhibition significantly attenuated the migratory and invasive capabilities of LUAD cells (Figure S9D,E). These in vitro observations were corroborated by in vivo metastasis models, where HMMR knockdown effectively suppressed lung metastasis formation (Figure 8L–O) and prevented distant metastasis to secondary organs including brain and bone (Figure 8P,Q). Thus, these comprehensive analyses across experimental platforms confirmed that HMMR functions as an important promoter of LUAD progression.

### 3.7 HMMR Overexpression Rescues the Suppressive Effects of CYYR1 Overexpression on LUAD Cell Phenotypes

To determine whether HMMR functions as a downstream mediator of CYYR1‐associated phenotypic regulation, transient rescue experiments were performed. A549 and H1299 cells were co‐transfected with CYYR1 and HMMR overexpression plasmids (Figure S10A). Transient restoration of HMMR expression partially reversed the suppressive effects of CYYR1 overexpression on LUAD cell proliferation (Figure S10B). Similarly, HMMR expression rescued the impaired colony formation ability in CYYR1‐overexpressing cells (Figure S10C). Cell cycle analysis further revealed that HMMR overexpression markedly reversed the CYYR1‐associated G1‐phase accumulation (Figure S10D).

In parallel, transwell and wound healing assays further indicated that HMMR overexpression significantly enhanced migratory and invasive capacities that were inhibited by CYYR1 (Figure S11A–C). These findings support HMMR as a functional downstream mediator of CYYR1‐associated tumor‐suppressive effects.

### 3.8 Multi‐Omics Integration and Experimental Analyses Identify CDC20 as a Functional Downstream Effector

To identify candidate downstream mediators associated with HMMR‐regulated LUAD progression, we performed label‐free quantitative proteomic analysis of HMMR‐knockdown cells, identifying 1937 candidate differentially abundant proteins according to the predefined exploratory fold‐change and nominal *p*‐value criteria (Table S6, Figure S12A–C).

Integration of this exploratory HMMR‐regulated proteomic profile with prognosis‐associated genes from the TCGA‐LUAD cohort narrowed the candidate pool to six genes, including HMMR itself (Figure S12D). After excluding HMMR, the remaining candidates were further prioritized using ten independent machine‐learning algorithms. Across all models, CDC20 consistently received the highest prioritization score among the evaluated candidates, suggesting its potential relevance in HMMR‐associated LUAD progression (Figure S13A,B). Subsequently, single cell and spatial transcriptome data showed that CDC20 is highly expressed in LUAD malignant cells (Figure S13C–E).

To experimentally determine whether CDC20 is regulated by HMMR, we examined CDC20 expression following genetic manipulation of HMMR. Although CDC20 did not remain statistically significant after multiple‐testing correction in the HMMR‐perturbation label‐free dataset, it was retained as a hypothesis‐generating candidate for subsequent validation based on the integrated prioritization analysis. Western blot analysis revealed that HMMR depletion significantly reduced CDC20 protein abundance in A549 and H1299 cells, whereas HMMR overexpression increased CDC20 protein levels (Figure S14A,B). These changes were consistently observed across independent LUAD cellular models, indicating that CDC20 expression is responsive to HMMR modulation. Together with the proteomic findings, these results support CDC20 as a functional downstream effector associated with HMMR‐mediated oncogenic regulation.

To further determine whether CDC20 mediates the biological effects of the CYYR1/HMMR axis, epistasis analyses were performed instead of conventional rescue experiments. In CYYR1‐restored LUAD cells, forced expression of CDC20 bypassed the inhibitory effects of CYYR1 restoration on cell proliferation, clonogenic capacity, and cell‐cycle progression (Figure S15). Similarly, CDC20 overexpression partially reversed the suppressive effects induced by HMMR depletion, restoring proliferative capacity and cell‐cycle progression in HMMR‐deficient cells (Figure S16). Moreover, CDC20 expression also bypassed the inhibitory effects of CYYR1 restoration or HMMR depletion on migration and invasion (Figures S17 and S18). Collectively, these findings support CDC20 as a functional effector downstream of the CYYR1/HMMR regulatory axis.

### 3.9 CYYR1 Restoration and Genistein Treatment Enhance Cisplatin Sensitivity in LUAD Models

Given that CYYR1 restoration suppressed malignant phenotypes and was associated with reduced HMMR/CDC20 pathway activity, we next investigated whether this regulatory vulnerability could be pharmacologically modulated to influence treatment responses. Because cisplatin (CDDP) remains a clinically relevant platinum‐based chemotherapy for LUAD, we first examined whether restoration of CYYR1 could influence cisplatin sensitivity.

Dose‐response analysis demonstrated that CYYR1 overexpression significantly increased the sensitivity of A549 cells to CDDP treatment, as evidenced by a reduced CDDP half‐maximal inhibitory concentration (IC50) compared with control cells (Figure 9A). These findings indicate that restoration of CYYR1 enhances cellular sensitivity to cisplatin treatment in LUAD cells.

**FIGURE 9:**
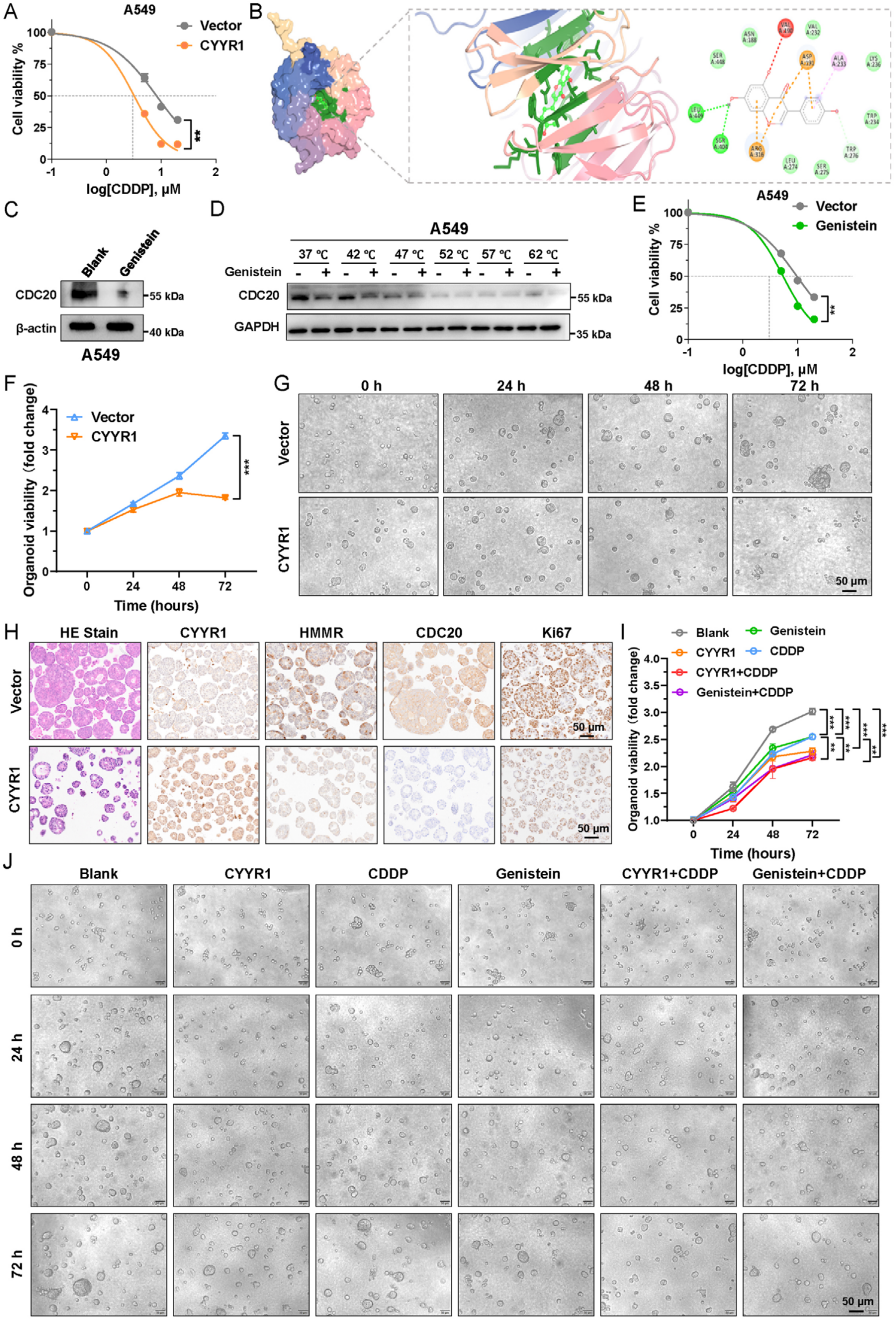
CYYR1 restoration and Genistein treatment enhance cisplatin sensitivity in LUAD models. (A) Dose‐response curves and half‐maximal inhibitory concentration (IC50) analysis of cisplatin (CDDP) in A549 cells with control vector or CYYR1 overexpression. CYYR1 restoration increased the sensitivity of LUAD cells to CDDP treatment. (B) Molecular docking analysis showing the predicted interaction between Genistein and CDC20. The three‐dimensional and two‐dimensional binding conformations of Genistein with CDC20 are shown, with the predicted binding free energy and interacting residues indicated. (C) Western blot analysis showing the effect of Genistein (50 µm) treatment on CDC20 protein abundance in A549 cells. (D) Cellular thermal shift assay (CETSA) showing increased thermal stability of CDC20 following Genistein treatment, providing cellular evidence consistent with CDC20 engagement or modulation. (E) Dose‐response curves and IC50 analysis of CDDP in A549 cells treated with vehicle control or Genistein (50 µm). Genistein treatment enhanced CDDP sensitivity in LUAD cells. (F, G) Quantitative analysis (F) and representative bright‐field images (G) showing reduced growth and viability following CYYR1 restoration in the tested patient‐derived LUAD organoid model. Scale bar: 50 µm. (H) Representative immunohistochemical staining of paraffin‐embedded LUAD organoid sections showing increased CYYR1 expression and decreased HMMR, CDC20, and Ki67 levels following CYYR1 restoration. Scale bar: 50 µm. (I) Viability analysis of the patient‐derived LUAD PDO model under the indicated treatment conditions, including control organoids, CYYR1‐overexpressing organoids, CDDP (10 µm), Genistein (50 µm), CYYR1 overexpression combined with CDDP (10 µm), and Genistein (50 µm) combined with CDDP (10 µm). (J) Representative bright‐field images of LUAD organoids under different treatment conditions. Scale bar: 50 µm. ^**^ *p* < 0.01, ^***^ *p* < 0.001.

Since CDC20 was identified as a functional downstream effector associated with the CYYR1‐HMMR regulatory pathway, we next explored the pharmacological effects of Genistein, a pleiotropic bioactive compound previously reported to influence CDC20‐associated cell‐cycle regulation [30]. Given that the specificity of Genistein toward CDC20 has not been established in LUAD, these experiments were designed as an exploratory assessment of whether Genistein treatment is associated with CDC20 modulation and altered cisplatin response.

Molecular docking analysis predicted a potential interaction between Genistein and CDC20, with a calculated binding free energy (ΔG) of −8.15 kcal/mol. The predicted three‐dimensional and two‐dimensional binding conformations of Genistein with CDC20 are shown in Figure 9B. The docking model predicted potential contacts involving ARG316, LEU449, SER404, TRP234, and VAL190, providing a hypothesis‐generating structural model rather than evidence of direct biochemical binding. Genistein treatment also reduced CDC20 protein abundance in A549 cells (Figure 9C). CETSA further demonstrated increased thermal stability of CDC20 following Genistein exposure (Figure 9D), providing cellular evidence consistent with CDC20 engagement or modulation following Genistein treatment. However, these findings do not establish direct biochemical binding or demonstrate that CDC20 is required for the pharmacological effects of Genistein.

We next evaluated whether Genistein could enhance cisplatin sensitivity in LUAD cells. Dose‐response analysis revealed that Genistein treatment decreased the IC50 value of CDDP in A549 cells compared with vehicle‐treated controls (Figure 9E). These findings demonstrate that Genistein treatment enhances cisplatin sensitivity in the tested LUAD cells. Although this effect occurred together with changes in CDC20 abundance and thermal stability, the present data do not establish that the enhanced cisplatin response is functionally mediated by CDC20.

To extend these observations to a patient‐derived model, we performed proof‐of‐concept experiments using one independent LUAD PDO line. Compared with control organoids, CYYR1‐overexpressing organoids exhibited reduced growth capacity and decreased viability (Figure 9F). Representative bright‐field images confirmed reduced organoid growth following CYYR1 restoration (Figure 9G). Immunohistochemical analysis demonstrated increased CYYR1 expression accompanied by decreased HMMR, CDC20, and Ki67 levels following CYYR1 restoration (Figure 9H), providing proof‐of‐concept evidence that the molecular and growth‐inhibitory effects associated with CYYR1 restoration are also detectable in this patient‐derived LUAD model.

Finally, we assessed whether CYYR1 restoration or Genistein treatment could influence cisplatin response in this PDO model. Compared with CDDP treatment alone, both CYYR1 overexpression combined with CDDP and Genistein combined with CDDP resulted in greater inhibition of organoid viability and reduced organoid growth (Figure 9I). Representative bright‐field images further confirmed the enhanced inhibitory effects of these combination treatments (Figure 9J). Collectively, these findings provide proof‐of‐concept patient‐derived evidence that CYYR1 restoration and Genistein treatment are each associated with enhanced cisplatin response in the tested LUAD PDO model. These results should not be interpreted as establishing inter‐patient generalizability, and the mechanistic contribution of CDC20 to the Genistein‐associated response remains to be determined.

## 4 Discussion

CYYR1 is a member of the single‐pass transmembrane protein family, and its functional research remains in the early stages [5, 6]. Recent studies have indicated that CYYR1 may have potential regulatory effects on ulcerative colitis and neonatal pulmonary hypoplasia [8, 9]. CYYR1 is also regarded as a key gene for discriminating chicken chromium poisoning and human corneal cell types [31, 32]. According to some clinical studies, CYYR1 DNA methylation can be used as a prognostic marker in human neuroblastoma [10]. However, the biological function of CYYR1 in cancer, particularly in LUAD, remains insufficiently understood. Recent studies have suggested that CYYR1 may participate in protein stability regulation through an adaptor‐like function [11]; however, whether CYYR1 regulates oncogenic protein homeostasis and LUAD progression remains unclear. Our study extends the current understanding of CYYR1 biology by demonstrating tumor‐suppressive effects following CYYR1 restoration in CYYR1‐deficient LUAD models and identifying its involvement in the regulation of oncogenic protein stability through a TRIM21‐dependent degradation mechanism.

We first delineated the clinical and biological significance of CYYR1 downregulation in LUAD. Our findings demonstrate that CYYR1 is significantly downregulated in LUAD tissues at both mRNA and protein levels, an observation corroborated by single‐cell and spatial transcriptomics. Importantly, low CYYR1 expression was associated with unfavorable patient prognosis, supporting its potential as a prognostic biomarker candidate. The prognostic relevance of CYYR1 was further supported by its prioritization within the multi‐gene machine‐learning framework. The epigenetic mechanism underlying this downregulation was identified as promoter hypermethylation, which was associated with reduced CYYR1 expression and could be partially reversed following DNA methyltransferase inhibitor treatment.

The central finding of our study lies in elucidating the molecular consequences of restoring CYYR1 expression in CYYR1‐deficient LUAD models. Our in vitro and in vivo experiments consistently demonstrate that CYYR1 restoration suppresses LUAD cell proliferation, migration, invasion, tumor growth, and metastatic progression. These findings establish the sufficiency of CYYR1 restoration to restrain malignant phenotypes in the experimental models examined, but do not by themselves establish the endogenous necessity of CYYR1 in LUAD cells retaining physiologically measurable expression. Employing an integrated proteomic strategy combining interactome screening with quantitative proteomics of CYYR1‐overexpressing cells, we identified HMMR as a candidate downstream mediator of CYYR1‐associated regulation. While HMMR has been implicated in tumor progression and recent studies have linked HMMR dysregulation to NSCLC metastasis [33], the mechanisms controlling HMMR protein stability in LUAD remain insufficiently characterized.

Our findings reveal that CYYR1 facilitates TRIM21‐dependent regulation of HMMR protein stability by promoting HMMR ubiquitination and proteasomal turnover. Rather than acting as an enzyme, CYYR1 appears to function as an upstream regulatory factor that facilitates productive TRIM21‐dependent regulation of HMMR stability. Although ectopic‐expression assays support associations among CYYR1, TRIM21, and HMMR, the present data do not establish the precise stoichiometry, topology, or spatial organization of a physiological endogenous ternary complex. This finding extends previous evidence suggesting that CYYR1 may participate in protein stability regulation [11] by demonstrating a previously unrecognized CYYR1‐associated mechanism controlling HMMR stability in LUAD.

The domain‐mapping results further suggest that the molecular organization of this regulatory machinery is unlikely to depend on a single binary interaction interface. CYYR1 preferentially associated with HMMR residues 365‐546, whereas TRIM21 interacted with multiple HMMR regions, and ubiquitination signals were detected across several HMMR fragments. These observations are consistent with a model in which TRIM21 can engage HMMR through multiple surfaces, while the 365‐546 region may provide a preferential CYYR1‐associated interface that facilitates productive substrate positioning or accessibility. Importantly, the current data do not establish that this region is uniquely or obligatorily required for CYYR1‐facilitated HMMR degradation.

TRIM21 has emerged as an important regulator of tumor‐associated protein homeostasis. Previous studies, including our previous work in NSCLC [29], have suggested that modulation of TRIM21 stability influences tumor progression. In the present study, we further establish the functional requirement of TRIM21 in CYYR1‐mediated HMMR regulation. Genetic depletion of TRIM21 substantially attenuated CYYR1‐associated HMMR degradation, reduced HMMR ubiquitination, and partially restored malignant phenotypes suppressed by CYYR1 restoration, supporting a functional requirement for TRIM21 in CYYR1‐mediated regulation of HMMR stability.

Nevertheless, because several biochemical interaction and ubiquitination analyses relied on ectopic expression systems, the present study does not establish the existence, stoichiometry, or spatial architecture of a fully physiological endogenous CYYR1‐TRIM21‐HMMR ternary complex. Endogenous CYYR1 abundance is extremely low in the LUAD models examined, which further limits reliable endogenous CYYR1 immunoprecipitation under the current experimental conditions. The precise endogenous organization of the CYYR1‐associated regulatory machinery and the physiological ubiquitination sites responsible for HMMR turnover therefore require further investigation using endogenous interaction approaches, proximity‐based interaction analyses, topology‐specific CYYR1 constructs, and site‐resolved ubiquitin profiling.

Beyond the CYYR1‐TRIM21‐HMMR regulatory module, our integrated multi‐omics analysis and functional experiments identified CDC20 as a downstream effector associated with HMMR‐mediated malignant progression. Functional epistasis analyses demonstrated that CDC20 overexpression partially bypassed the tumor‐suppressive effects induced by CYYR1 restoration or HMMR depletion, supporting its role as a downstream mediator of this regulatory pathway.

Although CDC20 represents an important downstream component of this regulatory network, our findings do not exclude contributions from additional cell‐cycle regulators, apoptotic pathways, senescence‐associated mechanisms, or stress responses that may collectively contribute to the biological effects of CYYR1 restoration.

Although CYYR1 is annotated as a single‐pass transmembrane protein, our data are consistent with the possibility that its intracellular region participates in regulation of cytoplasmic protein‐homeostasis machinery; however, this interpretation remains a working model rather than a directly mapped structural mechanism. The precise membrane topology, the contribution of the cytoplasmic region of CYYR1, and the exact subcellular site of CYYR1‐TRIM21‐HMMR regulatory machinery assembly remain to be experimentally defined using topology‐specific constructs, membrane fractionation, and organelle‐resolved analyses.

Given the identification of CDC20 as a functional downstream effector associated with CYYR1/HMMR signaling, we further explored the pharmacological effects of Genistein in LUAD. Previous studies have linked Genistein to cell‐cycle regulation, and our molecular docking analysis predicted a potential interaction with CDC20 [30]. Because molecular docking is hypothesis‐generating, however, this prediction alone cannot establish a direct Genistein–CDC20 interaction.

In the present study, Genistein treatment reduced CDC20 protein abundance and altered CDC20 thermal stability in CETSA assays, providing cellular evidence consistent with CDC20 engagement or modulation following Genistein exposure. Genistein also enhanced cisplatin sensitivity in LUAD cells and increased the inhibitory effect of cisplatin in the tested patient‐derived organoid model. Importantly, however, these pharmacological effects were not evaluated under genetic manipulation of CDC20, and therefore the present study does not establish that CDC20 is required for the Genistein‐associated enhancement of cisplatin response. Genistein is known to exert pleiotropic biological effects, and additional studies combining Genistein treatment with CDC20 loss‐ or gain‐of‐function approaches will be required to determine the specific contribution of CDC20 to this pharmacological phenotype.

Together, these observations provide preliminary pharmacological evidence that Genistein treatment is associated with CDC20 modulation and enhanced cisplatin response; however, they do not establish a CDC20‐dependent therapeutic mechanism.

From a signaling perspective, our work integrates these components into a coherent pathway: epigenetic silencing of CYYR1 reduces its ability to facilitate TRIM21‐dependent regulation of HMMR stability, resulting in HMMR accumulation and activation of CDC20‐associated oncogenic signaling, ultimately promoting LUAD progression.

While our study provides mechanistic and translational insights, several limitations and future directions remain. The structural organization of the CYYR1‐TRIM21‐HMMR regulatory machinery, including the precise interaction interfaces and ubiquitination sites responsible for HMMR turnover, requires further investigation through structural approaches, ubiquitin proteomics, and site‐directed mutagenesis. In addition, although the HMMR 365‐546 region showed preferential association with CYYR1 and relatively stronger ubiquitination signals, the functional necessity of this region has not been directly established. Domain‐specific deletion or mutagenesis together with site‐resolved ubiquitination analyses will be required to determine whether particular HMMR interfaces are preferentially required for CYYR1‐facilitated TRIM21‐dependent degradation.

A further limitation of the present study is that the functional analyses were predominantly performed using restoration‐based approaches in LUAD models with very low endogenous CYYR1 expression. Accordingly, our data establish the sufficiency of CYYR1 restoration to suppress malignant phenotypes but do not directly establish the endogenous necessity of CYYR1 for restraining HMMR/CDC20‐associated signaling. Consistent with our experimental observations, examination of the CCLE/DepMap dataset and the integrated Human Protein Atlas cell‐line resource indicated generally low or near‐undetectable CYYR1 transcript abundance across commonly used NSCLC/LUAD models, whereas relatively higher expression was observed predominantly in SCLC models [34, 35]. Future studies using LUAD models retaining physiologically measurable endogenous CYYR1 expression or endogenous genetic perturbation systems will be required to address this question.

A further limitation concerns the patient‐derived organoid experiments. All PDO analyses were performed using one independent LUAD organoid line, and therefore these experiments should be regarded as proof‐of‐concept patient‐derived evidence rather than broad translational validation. Although the observed effects of CYYR1 restoration on organoid growth and HMMR/CDC20 expression were consistent with findings from the cell‐line models, a single PDO cannot capture the molecular and therapeutic heterogeneity of LUAD across patients. Validation in additional independent PDO models representing diverse genomic backgrounds, endogenous CYYR1 expression levels, and CYYR1 methylation states will be required to determine the reproducibility and inter‐patient generalizability of these observations.

Furthermore, Genistein possesses pleiotropic biological activities, and although our docking, western blotting, and CETSA data are consistent with CDC20‐associated modulation, the present study does not establish that CDC20 is functionally required for the antitumor or cisplatin‐sensitizing effects of Genistein. Future studies combining Genistein treatment with genetic manipulation of CDC20 will be necessary to determine the relative contribution of CDC20 and other pathways to these pharmacological effects.

Collectively, our findings identify CYYR1 as an epigenetically dysregulated tumor‐suppressive regulator that influences oncogenic protein stability through a TRIM21‐dependent HMMR degradation pathway. The CYYR1‐TRIM21‐HMMR‐CDC20 axis represents a potential therapeutic vulnerability in LUAD, and strategies aimed at restoring CYYR1‐associated signaling or pharmacologically modulating downstream effectors warrant further investigation as potential approaches for improving treatment responses.

## 5 Conclusions

In summary, our study identifies CYYR1 as an epigenetically silenced tumor‐suppressive regulator in LUAD, with reduced expression associated with unfavorable patient prognosis. Mechanistically, we demonstrate that CYYR1 facilitates TRIM21‐dependent ubiquitination and proteasomal degradation of HMMR, thereby suppressing CDC20‐associated oncogenic signaling and LUAD progression. The CYYR1‐TRIM21‐HMMR regulatory relationship is supported by complementary biochemical, cellular, and genetic evidence, with in vivo models further supporting the functional effects of CYYR1 restoration and a patient‐derived LUAD organoid model providing proof‐of‐concept support. Moreover, CYYR1 restoration and Genistein treatment were associated with enhanced cisplatin sensitivity in the tested LUAD models, although the inter‐patient generalizability of the organoid findings and the specific contribution of CDC20 to the Genistein response remain to be established.

## Author Contributions

**Fengsheng Dai**, **Tingxiu Xiang**, **Dongxi Xiang**, **Yongzhong Wu**, and **Renyan Li** designed the study. Fengsheng Dai, **Jingyi Tang**, and **Huan Rong** performed the majority of the experimental work. **Yu Wang**, **Xiangyi Zhou**, **Can Xu**, and **Lili Li** performed bioinformatic analyses and data interpretation. Fengsheng Dai, **Fei Deng**, and **Xiao Yang** contributed to statistical analyses and manuscript drafting. **Dan Yang** and **Jianhua Wang** collected clinical samples. Tingxiu Xiang, Dongxi Xiang, and Yongzhong Wu provided study supervision and material support. Renyan Li, Lili Li, Dongxi Xiang, and Fengsheng Dai contributed to manuscript revision and refinement. All authors reviewed and approved the final manuscript.

## Funding

This study was supported by the National Natural Science Foundation of China (#82172619), the Natural Science Foundation of Chongqing (2025ZYZD004), the Medical and Industrial Integration Project (2022CDJYGRH‐002), the Science and Technology Research Program of Chongqing Municipal Education Commission (KJZD‐K202500108), Special Project for TechnologicalInnovation and Application Development of Chongqing (CSTB2024TIAD‐KJFZMSX0026), and the postdoctoral funding of Chongqing University Cancer Hospital (No. Y2024072).

## Ethics Statement

Histological diagnoses of the clinical specimens were confirmed by pathologists, and written informed consent was obtained from all participants for the collection and use of tissue specimens. The study involving human specimens was approved by the Ethics Committee of Chongqing University Cancer Hospital (approval number: CZLS2022030‐A‐5). All animal experiments were approved by the Animal Ethics Committee of Chongqing University Cancer Hospital (approval number: CQCH‐LAE‐A0000202089) and were performed in accordance with the approved institutional guidelines.

## Conflicts of Interest

The authors declare no conflicts of interest.

## Supporting information




**Supporting File 1**: advs77890‐sup‐0001‐SuppMat.docx.

**Supporting File 2**: advs77890‐sup‐0002‐SuppMat.xlsx.

## Data Availability

All publicly available datasets analyzed in this study were obtained from TCGA and GEO databases, and accession numbers are provided in the Methods section. Processed quantitative proteomic datasets generated in this study are included in the Supplementary Information. Raw mass spectrometry data can be made available upon reasonable request subject to institutional policies.
