# Supplementary material for: Epigenetic Silencing of CYYR1 Promotes Lung Adenocarcinoma Progression Through TRIM21‐Dependent Regulation of HMMR Stability
Source: Adv Sci (Weinh). 2026 Oct 11:e77890. Online ahead of print. doi: 10.1002/advs.77890 (PMC13654864; doi:10.1002/advs.77890)
Supplement: Supplementary file 1 [file ADVS-9999-e77890-s001.docx]

**Epigenetic silencing of CYYR1 promotes lung adenocarcinoma progression through TRIM21-dependent regulation of HMMR stability**

**Figure S1. Single-cell and spatial transcriptomic analyses delineate the tumor microenvironment and CYYR1 expression patterns in public NSCLC reference datasets.**

(A) UMAP reduced-dimension visualization of single-cell transcriptome sequencing data of LUAD samples. (B) Bubble plot showing the expression of key marker genes defining the major cell clusters identified in the single-cell data. (C) Analysis of major cellular compartments across LUAD spatial transcriptomic datasets. (D) Spatial distribution of CYYR1 expression across LUAD tissue regions.

**Figure S2** **Pan-cancer analysis of CYYR1 methylation in TCGA cohorts.** ns *P* > 0.05, * *P* <0.05, ** *P* <0.01, *** *P* <0.001.

**Figure S3. CYYR1 is frequently hypermethylated at its promoter region in LUAD.**

(A) MSP analysis of CYYR1 promoter methylation status in an independent cohort of LUAD tissues (n = 192). (B) Schematic of the CpG islands within the CYYR1 promoter region.

**Figure S4. Validation of the tumor-suppressive effects of CYYR1 restoration in genetically distinct LUAD models.**

(A) Validation of CYYR1 overexpression efficiency in H2122 and PC-9 cells by RT-qPCR and western blotting. (B) CCK-8 assays showing the effects of CYYR1 overexpression on cell viability in H2122 and PC-9 cells. (C) Colony formation assays evaluating the impact of CYYR1 restoration on the clonogenic capacity of H2122 and PC-9 cells. (D-E) Transwell assays showing the effects of CYYR1 overexpression on cell migration (D) and invasion (E) abilities in H2122 and PC-9 cells. (F) Wound healing assays demonstrating the effects of CYYR1 restoration on two-dimensional migratory capacity of H2122 and PC-9 cells. * *P* <0.05, ** *P* <0.01, *** *P* <0.001.

**Figure S5. Identification of HMMR as a CYYR1-associated effector in LUAD.**

(A) Venn diagram of the screened CYYR1-interacting proteins. (B-C) KEGG enrichment analysis of interacting proteins (B) and GO enrichment analysis (C). (D) Volcano plot of the TMT-based proteomic analysis comparing CYYR1-overexpressing cells with control cells, showing candidate differentially abundant proteins identified according to the predefined exploratory fold-change and nominal P-value criteria. FDR-adjusted P values are provided in Table S5. (E-F) KEGG pathway enrichment (E) and GO term enrichment (F) analysis of the differentially expressed proteins from the proteomic data.

**Figure S6. Multi-modal prioritization and validation identifies HMMR as a key CYYR1-associated effector in LUAD.**

(A) Residual distribution boxplot for 10 different machine learning models during cross-validation, used for feature selection. (B) Variable importance ranking plot across the 10 machine learning methods, highlighting the top candidate (HMMR). (C) A violin plot depicting the expression of HMMR in normal epithelial cells versus tumor epithelial cells. (D) Spatial distribution of HMMR expression across distinct microscopic regions on the LUAD spatial transcriptomics slice. (E) Bar graph quantifying HMMR expression levels in malignant, mixed malignant, and normal regions defined by pathological annotation on the spatial transcriptomics data.

**Figure S7. Bioinformatic prediction of HMMR post-translational modification sites and computational structural modeling of CYYR1-, TRIM21-, and HMMR-associated interactions.**

(A) Bioinformatics analysis using established protein post-translational modification (PTM) databases to catalog and display known and predicted modification sites (such as phosphorylation, ubiquitination) on the HMMR protein. (B) Computational structural modeling illustrating predicted interaction configurations between HMMR and TRIM21, HMMR and CYYR1, and a putative multi-protein arrangement involving CYYR1, TRIM21, and HMMR. These models are hypothesis-generating and do not establish the existence, stoichiometry, or physiological architecture of an endogenous CYYR1-TRIM21-HMMR ternary complex.

**Figure S8. TRIM21 depletion attenuates CYYR1-mediated suppression of LUAD malignant phenotypes.**

(A) CCK-8 assays showing cell viability of A549 and H1299 cells with CYYR1 overexpression alone or combined with TRIM21 knockdown. (B) Colony formation assays evaluating the effects of CYYR1 restoration and TRIM21 depletion on clonogenic capacity. (C-D) Transwell assays showing migration (C) and invasion (D) capacities under indicated conditions. (E) Wound healing assays evaluating two-dimensional migratory capacity following CYYR1 overexpression with or without TRIM21 knockdown. Data are presented as mean ± SD. Statistical analyses were performed using one-way ANOVA with appropriate post hoc comparisons. ** *P* <0.01, *** *P* <0.001.

**Figure S9. HMMR knockdown suppresses LUAD cell proliferation, migration, and invasion.**

(A) CCK-8 assays evaluating cell proliferation following HMMR knockdown. (B) Colony formation assays assessing clonogenic capacity. (C) Flow cytometric analysis of cell-cycle distribution following HMMR depletion. (D) Transwell migration and invasion assays. (E) Wound-healing assays evaluating two-dimensional migratory capacity at the indicated time points. ns *P* >0.05, * *P* <0.05, ** *P* <0.01, *** *P* <0.001.

**Figure S10. Transient HMMR restoration partially reverses the suppressive effects of CYYR1 overexpression on LUAD cell proliferation.**

(A) Detection of cell transfection efficiency by western blotting. (B-C) CCK-8 (B) and colony formation (C) assays were used to detect the proliferation of LUAD cells following co-transfection of HMMR and CYYR1 overexpression plasmids. (D) Flow cytometric analysis of cell-cycle distribution following co-expression of CYYR1 and HMMR. ns *P* >0.05, ** *P* <0.01, *** *P* <0.001.

**Figure S11. HMMR overexpression mitigates the effects of CYYR1 on LUAD cell migration and invasion.**

(A-C) After co-transfection of CYYR1 and HMMR overexpression plasmids into A549 and H1299 cells, the migration and invasion of LUAD cells were detected by Transwell assays (A-B) and wound healing tests (C). ** *P* <0.01, *** *P* <0.001.

**Figure S12. Multi-omics identification of CDC20 as a candidate downstream effector of HMMR in LUAD.**

(A) Volcano plot from the label-free quantitative proteomic analysis comparing HMMR-knockdown LUAD cells with shRNA control cells, showing candidate differentially abundant proteins identified according to the predefined exploratory fold-change and nominal P-value criteria. FDR-adjusted P values are provided in Table S6. (B-C) KEGG pathway (B) and GO term (C) enrichment analyses of the candidate differentially abundant proteins identified in the HMMR-perturbation proteomic dataset. (D) Venn diagram integrating the exploratory HMMR-regulated proteomic profile, CYYR1-associated proteomic changes, and prognosis-associated genes from the TCGA-LUAD cohort, yielding a set of candidate downstream genes for subsequent prioritization.

**Figure S13. Multi-modal prioritization and validation of CDC20 as a downstream effector of HMMR in LUAD.**

(A) Residual distribution boxplot for 10 different machine learning models during cross-validation, used for feature selection. (B) Variable importance ranking plot across the 10 machine learning methods, highlighting the top candidate (CDC20). (C) A violin plot depicting the expression of CDC20 in normal epithelial cells versus tumor epithelial cells. (D) Spatial distribution of CDC20 expression in the analyzed NSCLC spatial transcriptomic dataset. (E) Quantification of CDC20 expression across malignant, mixed malignant, and normal spatial regions.

**Figure S14. HMMR modulates CDC20 protein abundance in LUAD cells.**

(A-B) Western blot analysis of CDC20 protein expression in A549 (A) and H1299 (B) cells following HMMR knockdown using shRNA or HMMR overexpression. HMMR depletion reduced CDC20 protein abundance, whereas HMMR restoration increased CDC20 expression.

**Figure S15. CDC20 overexpression attenuates the suppressive effects of CYYR1 overexpression on LUAD cell proliferation.**

(A) Western blot analysis confirming the overexpression efficiency of CYYR1 and CDC20 following plasmid transfection. (B-C) Cell proliferation was assessed by CCK-8 assay (B) and colony formation assay (C) in LUAD cells co-transfected with CYYR1 and CDC20 overexpression plasmids. (D) Flow cytometry-based cell-cycle analysis showing that CDC20 overexpression partially reverses the CYYR1-associated increase in G1-phase accumulation. ns *P* >0.05, ** *P* <0.01, *** *P* <0.001.

**Figure S16. CDC20 overexpression attenuates the suppressive effects of CYYR1 overexpression on LUAD cell migration and invasion.**

(A-C) Transwell assays (A-B) and wound healing assays (C) were performed in A549 and H1299 cells following co-transfection with CYYR1 and CDC20 overexpression constructs to evaluate migratory and invasive capacities. ** *P* <0.01, *** *P* <0.001.

**Figure S17. CDC20 overexpression partially reverses the suppressive effects of HMMR depletion on LUAD cell proliferation.**

(A) Western blot analysis validating the depletion efficacy of HMMR and the overexpression of CDC20 following plasmid transfection. (B-C) Proliferative capacity was evaluated in LUAD cells upon co-expression of CDC20 in the context of HMMR knockdown, using CCK-8 (B) and colony formation (C) assays. (D) Cell-cycle distribution was analyzed by flow cytometry, showing that CDC20 overexpression partially reverses HMMR depletion-associated alterations in cell-cycle distribution, including increased G1-phase accumulation. * *P* <0.05, ** *P* <0.01, *** *P* <0.001.

**Figure S18. CDC20 overexpression partially reverses the impaired migration and invasion associated with HMMR depletion in LUAD cells.**

(A-C) Transwell migration (A), invasion (B), and wound healing (C) assays assessed the migratory and invasive capacities of A549 and H1299 cells. Overexpression of CDC20 reversed the suppression of migration and invasion caused by HMMR knockdown. ** *P* <0.01, *** *P* <0.001.
